# Ag‐Decorated Hydrogen Molybdenum Bronze Nanotubes as Dual‐Action Agents Against *Bacillus subtilis*: Experimental and Theoretical Insights Into Membrane Damage and Protein Interference

**DOI:** 10.1155/bca/9270509

**Published:** 2026-02-24

**Authors:** Shabnam Yavari, Neda Eghtesadi, Kayode Olaifa, Darya Shafiee, Amir H. Montazer, Reza Rasuli, Ebrahim Nemati-Kande, Forough Pakzadi, Sorour Faramarzi, Mehdi Shafiee

**Affiliations:** ^1^ Department of Electrical and Computer Engineering, School of Engineering and Digital Sciences, Nazarbayev University, Astana, 010000, Kazakhstan, nu.edu.kz; ^2^ Energetic Cosmos Laboratory, Nazarbayev University, Astana, 010000, Kazakhstan, nu.edu.kz; ^3^ Department of Physics, Faculty of Science, University of Zanjan, Zanjan, Iran, znu.ac.ir; ^4^ Biofilm Laboratory, Department of Chemical and Materials Engineering, School of Engineering and Digital Sciences, Nazarbayev University, Astana, 010000, Kazakhstan, nu.edu.kz; ^5^ Department of Biology, Nazarbayev Intellectual School of Biology and Chemistry, Aktau, 130000, Kazakhstan; ^6^ Department of Biomedical Sciences, School of Medicine, Nazarbayev University, Astana, 010000, Kazakhstan, nu.edu.kz; ^7^ Department of Medical Equipment Technology Engineering, Al-Hadba University, Mosul, Iraq; ^8^ Department of Physical Chemistry, Faculty of Chemistry, Urmia University, Urmia, 5756151818, Iran, urmia.ac.ir; ^9^ Research Center for Computational and Theoretical Molecular Engineering (RC2TME), Khazar University, Baku, 1009, Azerbaijan, khazar.org; ^10^ Department of Biology, Faculty of Science, Urmia University, Urmia, 5756151818, Iran, urmia.ac.ir

**Keywords:** antibacterial, *Bacillus subtilis*, biofilms, hydrogen molybdenum bronze, nanotubes, silver NPs

## Abstract

Bacterial biofilms are a persistent challenge in industrial settings such as water treatment and food processing, contributing to antimicrobial resistance, operational inefficiencies, and environmental burden. Here, we report on the synthesis and multiscale evaluation of hydrogen molybdenum bronze nanosheets (HMB‐NSHs) and their silver‐decorated nanotube derivatives (Ag–decorated HMB‐NTs), produced via an arc discharge method. High‐resolution structural analyses revealed crystalline, ultrathin HMB sheets and tubular architectures adorned with uniformly distributed Ag nanoparticles (∼3–5 nm). While HMB‐NSHs were biologically inert, Ag–decorated HMB‐NTs demonstrated potent antibacterial effects against *Bacillus subtilis*, inhibiting planktonic growth (75.7%), biofilm formation (77.7%), and biofilm eradication (64.3%) at 25 μg/mL. Complementary SEM and fluorescence microscopy visualizations revealed pronounced morphological membrane damage such as wrinkling, roughening, and biofilm reduction signatures absent in control and HMB‐treated samples, facilitating metal ion deposition and localized oxidative stress. At the molecular level, multiscale computational modeling, including molecular docking, DFT, QTAIM, RDG, and IGM analyses, provided atomic‐resolution insights into dual‐site antibacterial action. The Ag and HMB moieties interact favorably with both the cell‐wall penicillin‐binding protein (PDB ID: 4WO7) and intracellular division regulator FtsZ (PDB ID: 2VAM), forming energetically stable complexes. QTAIM metrics confirmed extensive van der Waals and hydrogen bonding networks with 4WO7, whereas RDG and IGM surfaces visualized dense noncovalent contact regions. Ag–FtsZ interactions, though weaker, suggest possible disruption of cell cycle machinery upon internalization. These findings establish Ag‐decorated HMB‐NTs as a dual‐function nanomaterial: HMB scaffolds promote surface adhesion and stability, whereas Ag enables membrane destabilization and intracellular disruption. Together, these processes highlight membrane damage and protein interference as the primary antibacterial mechanisms, underscoring their potential as a next‐generation antibacterial platform, particularly against biofilm‐forming and industrially relevant bacteria such as *Bacillus subtilis*.

## 1. Introduction

The world is confronting major challenges spanning environmental sustainability, energy security, and public health [1–3]; among these, bacterial infections remain a formidable and urgent global health threat [4]. The impact of these infections goes beyond the confines of medical concerns, exerting a substantial burden on public health, medicine, and the global economy [5]. Antibiotic therapy remains a potent weapon in combating bacterial infections. However, the misuse and overuse of antibiotics have led to antimicrobial resistance among bacterial pathogens, increasing the risk of infection recurrence and patient mortality [6]. Moreover, the adaptability of bacteria and the persistent misuse of antibiotics in healthcare settings have considerably compromised their effectiveness [7]. Consequently, urgent actions and innovative approaches are imperative to tackle this escalating public health concern. Given the escalating challenges posed by antibiotic resistance in bacterial infections, it is imperative to explore alternative and innovative approaches to combat these pathogens effectively [8]. This urgency has fueled the development of nanotechnology, driving the discovery of advanced antimicrobial drugs and cutting‐edge strategies [4].

Nanomaterials, with their unique physical and chemical properties, offer promising avenues for antibacterial interventions, directly combating and eliminating bacteria [9–11]. Carbon‐based materials, inorganic metals (Ag, Mo, Cu, etc.), polymers, metal oxides, nanomaterials, and their nanocomposites have been prominently employed in antibacterial fields [12], demonstrating unique properties and functionalities that make them effective in combating bacterial infections and developing innovative antimicrobial strategies. In addition, the synthesis of nanomaterial composites provides excellent antibacterial properties. Silver nanoparticles (Ag NPs) hold the foremost position among nanomaterials extensively employed in modern healthcare. Research focused on these NPs has revealed their remarkable antibacterial potency against a diverse array of both Gram‐positive and Gram‐negative bacteria, including those that manifest multidrug resistance [13]. It has been established that Ag^+^ ions possess the capability to disrupt microbial cell walls, denature cellular proteins, hinder cell respiration, and ultimately trigger cellular demise [14, 15]. The antibacterial efficacy of Ag NPs is significantly influenced by their size; smaller particles tend to exhibit heightened effectiveness against microorganisms [16, 17]. Conversely, when the size of Ag NPs diminishes, they tend to aggregate more readily, thereby compromising their chemical attributes and diminishing their antibacterial prowess [18]. To address this, efforts have been made to bolster the properties of Ag NPs by coupling them with other substances such as TiO_2_ [19], ZnO [20], MoO_3_ [21], and carbon nanotubes (NTs) [22].

In the realm of antibacterial research, molybdenum (Mo)–based nanomaterials have garnered significant interest due to their exceptional physicochemical characteristics. The importance of molybdenum as an essential trace element holds significant implications for various professional fields, such as agriculture [23], microbiology [24], and veterinary sciences. Its vital role in supporting the growth and health of microorganisms, plants, and animals, through involvement in metabolic processes and enzymatic functions, underscores its indispensability within biological systems [25]. Interestingly, it is worth mentioning that molybdenum‐based materials possess favorable characteristics, including biocompatibility, near‐infrared absorption capabilities, and biodegradability, rendering them highly suitable candidates for antibacterial research [26]. Molybdenum‐based nanomaterials have shown great promise in a wide range of applications across different fields, including electronics [27], energy storage [28], catalysis [29], antibacterial applications [30], and biomedical imaging [31].

Molybdenum bronze NPs, alternatively referred to as Y_x_MoO_x_ NPs, encompass a distinctive and multifaceted compound within the realm of transition metal oxides [32]. Here, the variable “Y” can encompass elements such as H, Li, K, Na, and Rb. Chemically, hydrogen molybdenum bronze (HMB) aligns itself as a member of the molybdenum bronze family, denoted by the formula H_x_MoO_3_ [32, 33]. Within this formula, the variable “x” signifies the quantity of hydrogen atoms that can integrate into its intricate crystal lattice structure. The intercalation process involves the insertion of hydrogen atoms between the layers of molybdenum oxide, leading to remarkable changes in its physical and electronic properties [34]. Researchers are continuously engaged in exploring pioneering synthesis methodologies, meticulously scrutinizing the diverse attributes of HMB to unlock its full potential [35–37]. Despite its innovative nature, current research into its attributes, particularly in relation to antibacterial properties, remains limited. Notably, within the broader spectrum of molybdenum bronzes, only a singular study has focused on sodium molybdenum bronze, probing its antibacterial behavior [30].

In this study, our primary emphasis centers on the development of a novel and cost‐effective synthesis method of HMB, while also upholding its high purity. Subsequently, our endeavor extends to enhancing its antibacterial characteristics through the manipulation of morphology and the incorporation of Ag NPs. Moreover, given molybdenum’s established utility in soil applications, we explore its potential for bacterial studies. On the other hand, we theoretically investigate the antibacterial activity of Ag–decorated HMB‐NTs based on different computational methods, providing insights into membrane damage and protein interference. The inherent molybdenum composition presents the prospect of pioneering a groundbreaking new material with several synergistic advantages. Looking ahead, a promising avenue for exploration involves synergistically amalgamating the intrinsic photonic properties of these NPs with their antibacterial efficacy in soil. This collaborative approach may herald the development of antibacterial soil capable of harnessing sunlight to augment its antibacterial properties, paving the way for innovative solutions at the intersection of materials science and environmental health.

## 2. Materials and Methods

### 2.1. Synthesis of HMB‐NSHs and Ag‐Decorated HMB‐NTs

An arc discharge method was used to synthesize HMB‐NSHs and Ag‐decorated HMB‐NTs. Two molybdenum metal rods were used as electrodes (10 cm in length and 2 mm in diameter) with a purity of 99.99%. The welding machine (Resanta Company) was used as a dependable power source. Silver nitrate (AgNO_3_, Merck, Germany) and deionized (DI) water (200 mL) were used.

For the synthesis of HMB‐NSHs, molybdenum rods were connected to the electrodes of the welding machine and the electrode assembly was placed in a beaker with DI. A current of 30 A was applied to both ends of the electrodes, and plasma was produced. We put both ends of the electrodes in water to form HMB in the form of NSHs. These NSHs were dispersed in water, and as a result, the solution was colored blue. The plasma‐assisted process was repeated several times while ensuring that the solution temperature remained below 80°C. Finally, the solution containing HMB‐NSHs was poured into the watch glass and dried in the oven at 80°C.

AgNO_3_ solution with a concentration of 10^−3^ M was prepared. Next, 40 mg of dried HMB‐NSHs was added to 100 mL of DI water in a beaker placed on a heater and stirred at 200 rpm. The NSHs were allowed to obtain a homogeneous dispersion at 70°C for 30 min. Simultaneously, the AgNO_3_ solution was added to the HMB‐NSHs, and the mixture was placed under UV irradiation for 5 h to facilitate the reaction. The AgNO_3_ and HMB solutions were prepared with a molybdenum to nitrate ratio of 5.2:1. During this step, the solution changed color from blue to brown. The final solution was dried at 200°C on a hot plate to obtain Ag‐decorated HMB‐NTs.

### 2.2. Planktonic Growth

Different concentrations of HMB‐NSHs and Ag‐decorated HMB‐NTs were evaluated on the planktonic growth of *B. subtilis* in 48‐well CellBIND® tissue culture plates, flat bottom, clear, sterile, with lid (Corning Inc., Corning, NY, USA), Gen5™ Microplate Reader and Imager Software (BioTek Instruments). All the bacterial growth experiments were performed using Luria–Bertani (LB) medium, while growth was monitored through intermittent measurement of absorbance at 600 nm (OD_600_) using a Gen5™ Microplate Reader and Imager Software coupled with a microplate reader (BioTek Instruments, Winooski, VT, USA), over a duration of 48 h. Three independent biological replicates were used, and values are reported in the form of average ± standard deviation. The HMB‐NSHs and Ag‐decorated HMB‐NTs concentrations ranged from 5 μg/mL to 1000. In each experiment, the added components were dissolved in the prepared LB medium and then sterilized through filtration using a 0.2‐μm syringe filter (Merck Millipore, Burlington, MA, USA). Each well of the 48‐well plate was filled with 800 μL and incubated at 37°C. For inoculation, an overnight culture grown in 50‐mL volume‐sterilized tubes with caps closed at 37°C, under shaking conditions (180 rpm), was used as inoculum. The wells were inoculated with an initial OD_600_ = 0.1, equivalent to 10^6^ colony‐forming units per mL (CFU/mL). For each experimental condition, a minimum of four independent biological replicates were used.

### 2.3. Biofilm Formation Assay

At different concentrations, the crystal violet assay was employed to investigate the effect of HMB‐NSHs and Ag‐decorated HMB‐NTs on the inhibition of biofilm growth. The crystal violet assay was employed as a quantitative method to investigate biofilm formation on the walls and bottom of a microtiter plate. Briefly, *B. subtilis* was cultivated for 48 h in 48‐well plates (Corning Inc., Corning, NY, USA) at 37°C in the presence of varying concentrations of HMB‐NSHs and Ag‐decorated HMB‐NTs with an inoculum of initial OD_600_ adjusted to 0.1. Following the 48‐h incubation period, the wells were gently emptied of any unattached cells and media, and the microtiter plates were air‐dried for 15–20 min at room temperature. Next, 1000 μL of 0.1% crystal violet solution (Crystal Violet, ACROS organics; Geel, Belgium, Code: 405830250) was pipetted into each well. Thereafter, the plates were left undisturbed for 30 min. Subsequently, 33% (v/v) acetic acid was applied, and the solubilized crystal violet was transferred to a new flat‐bottomed microtiter dish. Biofilm estimate was quantified by optical density measurement at 570 nm using the Gen5™ Microplate Reader and Imager Software (BioTek Instruments, Winooski, VT, USA). For each experimental condition, a minimum of four independent biological replicates were used.

### 2.4. Biofilm Removal Assay

The effect of HMB‐NSHs and Ag‐decorated HMB‐NTs on preformed biofilms was evaluated using the crystal violet staining method. *B. subtilis* cultures were initially grown in 48‐well tissue culture plates (Corning Inc., Corning, NY, USA) under static conditions at 37°C for 48 h to allow biofilm formation. Following incubation, the culture medium was gently removed, and wells were washed carefully to eliminate nonadherent cells. Fresh LB medium containing different concentrations of HMB‐NSHs and Ag‐decorated HMB‐NTs was added to the wells containing preformed biofilms. The plates were then incubated for an additional 24 h at 37°C. After treatment, biofilm biomass was quantified using the same crystal violet staining procedure described in the biofilm formation assay section. All experiments were performed with at least four independent biological replicates.

### 2.5. Computational Methods

#### 2.5.1. Molecular Docking Computations

AutoDock 4.2 software [38] was used to investigate the interactions of Ag and HMB with *Bacillus subtilis*, focusing on two protein structures with PDB IDs: 4WO7 (cell wall) and 2VAM (cell cycle). A grid encompassing the entire protein structure with a spacing of 0.944 Å was generated to analyze the binding sites of the compounds. The Lamarckian genetic algorithm (LGA) was employed to predict their binding modes. Key parameters included an initial population size of 150, 100 genetic algorithm (GA) runs, and 27,000 generations per docking simulation. All other parameters were kept at their default values. Postdocking, the interactions between the compounds and the bacterial structure—particularly amino acid residues within a 3 Å radius—were analyzed using the VMD [39] visualization tool.

#### 2.5.2. Density Functional Theory (DFT) Calculation

To gain a detailed atomic‐level understanding of the interactions between *Bacillus subtilis* proteins and the Ag and HMB ligands, quantum mechanical simulations were performed using DFT [40]. Active binding sites, identified through molecular docking, were isolated and refined using LigPlot+ [41], a specialized tool for visualizing protein–ligand interactions. To ensure electronic closure and chemical stability, terminal atoms at these extracted sites were capped with hydrogen atoms. Subsequent quantum chemical calculations employed B3LYP hybrid functional with the 6‐311++G(d,p) basis set, as implemented in Gaussian 16 [42]. The resulting electronic wave functions were further analyzed using the quantum theory of atoms in molecules (QTAIM) [43], a topological approach introduced by Bader and MacDougall [44–46], which characterizes interatomic interactions through electron density distribution analysis.

To complement the topological findings and provide a more comprehensive assessment of weak intermolecular forces, particularly van der Waals (vdW) interactions and hydrogen bonding, additional analyses were conducted using the independent gradient model (IGM) [47] and reduced density gradient (RDG) [48] methods. These calculations were performed using the Critic2 software suite [49], designed for real‐space electron density analysis. Molecular geometries and interaction surfaces were visualized using VMD [39].

### 2.6. Materials Characterization and Testing

#### 2.6.1. Fourier Transform Infrared Spectroscopy (FTIR) and UV–Visible Spectrophotometry Analysis

The interaction of Ag‐decorated HMB‐NTs with bacterial cell walls was studied using FTIR. A volume of 15 mL of bacterial cell suspension was treated with 50 μg/mL Ag‐decorated HMB‐NTs for 24 h along with negative control (untreated bacterial suspension). The samples were dried off in the oven at 25°C, and the collected pellets were washed with 1XPBS three times. The dried powder was then subjected to FTIR using FTIR Nicolet iS10 FT‐IR Spectrometer equipped with a ZnSe flat crystal. Spectra were determined by recording 32 transmission scans in the range of 4000–400 cm^−1^ with a resolution of 4 cm^−1^. The scan graphs were collected using OMNIC software. For UV spectroscopy analysis, we employed the Shimadzu UV 2600‐i UV–Vis spectrophotometer to investigate the optical properties of the HMB‐NSHs and Ag‐decorated HMB‐NTs in depth.

#### 2.6.2. Microscopy

In this section, we detail the sample preparation procedures for high‐resolution transmission electron microscopy (HRTEM; JEOL JEM‐1400 Plus) analysis of NPs using copper mesh grids. HMB‐NSHs and Ag‐decorated HMB‐NTs were dispersed in DI water to prevent aggregation, followed by the deposition of a drop of the NSHs and NTs suspension onto precleaned, holey carbon‐coated copper grids. DI water evaporation was conducted to ensure the even distribution of HMB‐NSHs and Ag‐decorated HMB‐NTs across the grid. Subsequently, HRTEM grids were loaded onto the microscope holder for high‐resolution imaging, allowing for the comprehensive characterization of NSHs and NTs morphology, size distribution, and crystallography. In addition to the HRTEM analysis, energy‐dispersive X‐ray spectroscopy (EDS) was performed in a scanning electron microscope (SEM JEOL; JSM‐IT200) equipped with an EDS detector. A full (survey) spectrum was first acquired to identify the elements present based on their characteristic X‐ray lines. For elemental mapping, the electron beam was scanned over the selected field of view, and the X‐ray intensity corresponding to each characteristic line (e.g., O‐K, Mo‐L, Ag‐L) was recorded pixel by pixel to generate 2D distribution maps. It should be mentioned that the dried HMB‐NSHs and Ag‐decorated HMB‐NTs samples were coated with a thin layer of gold to enhance conductivity. These maps provide qualitative/semiquantitative information on elemental homogeneity and colocalization.

The formation of biofilm at the bottom of the ibidi® 8‐well glass bottom chamber was observed using Axio Zoom V16, Carl Zeiss microscopy. *B. subtilis* treated with different concentrations of HMB‐NSHs and Ag‐decorated HMB‐NTs were cultivated in an 8‐well glass ibidi μ‐slide for 48 h. Following the incubation period, the wells were gently emptied of any unattached cells and media, and the glass slide was air‐dried for 15–20 min at room temperature. Next, 800 μL of 0.1% crystal violet solution was added to each well, and the plates were incubated at room temperature for 10–15 min. Subsequently, the ibidi® chamber was immersed gently in distilled water to remove the excess crystal violet and air‐dried. The stained biofilm was then observed at varying magnifications using Axio Zoom V16, Carl Zeiss microscopy (Carl Zeiss, Germany).

The preparation of SEM samples for bacteria‐containing specimens was conducted prior to their analysis. To begin, the selected samples were incubated in a 2% formaldehyde solution for a duration of 20 min. This step was followed by postconfirmation, involving the immersion of the samples in a 1% weight osmium tetroxide solution for approximately 1 h. Next, the samples underwent dehydration through a series of ethanol solutions with concentrations of 70%, 90%, 95%, and 100% weight, respectively. Each ethanol immersion lasted for nearly 10 min. Finally, the samples were air‐dried for approximately 15 min at room temperature. To enhance their conductivity, a thin layer of gold, approximately 10 nm in thickness, was deposited onto the dried samples.

#### 2.6.3. X‐Ray Photoelectron Spectroscopy (XPS) and X‐Ray Diffraction (XRD)

In the preparation section for XPS and XRD, a series of steps were undertaken to ensure accurate analysis of the HMB‐NSHs and Ag‐decorated HMB‐NTs. For XPS, the samples were initially placed in a high‐vacuum chamber to remove any surface contaminants. Subsequently, monochromatic X‐rays were directed at the sample, causing the emission of photoelectrons. The emitted electrons were then analyzed to determine the elemental composition and chemical states of the material. The procedure was executed to eliminate any potential sources of contamination, ensuring a precise representation of the surface chemistry of the NSHs and NTs. Afterward, the measurements were performed using the Thermo Scientific XPS. Notably, this instrument employs a monochromated low‐power Al Ka X‐ray source with an energy level of 1486.6 eV. For XRD, the NSHs and NTs were ground into a fine powder, loaded onto a single‐crystal silicon holder, and X‐rays were directed at the sample at a fixed angle. SmartLab was employed for precise measurements, and the data were analyzed using Xpert software.

## 3. Results and Discussion

### 3.1. UV–Vis Results

**FIGURE 1:**
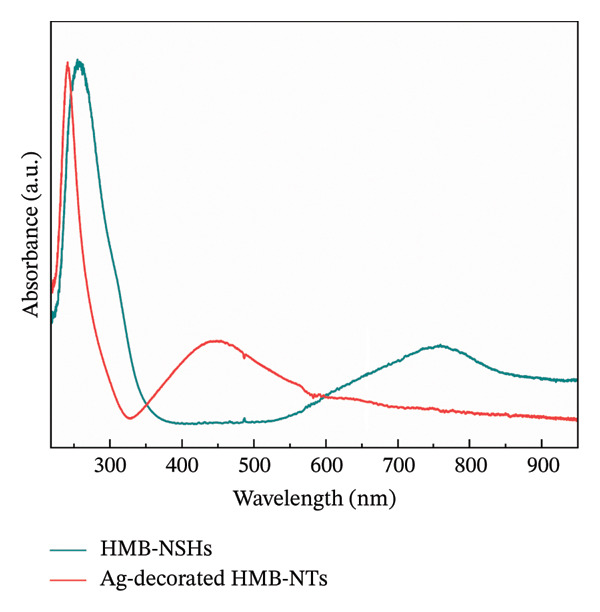
Optical absorption spectra of HMB‐NSHs and Ag‐decorated HMB‐NTs.

The optical absorption spectra of the blue HMB, both before and after being mixed with the AgNO_3_ solution, are presented in Figure 1. A significant absorption peak is observed in the range of 650–850 nm for the blue NSHs (Figure 1) [50], which provides direct evidence of localized surface plasmon resonance. The blue color of the HMB‐NSHs arises from the intervalence charge‐transfer transition between Mo^V^ and Mo^VI^ coexisting within them [51]. Upon the addition of AgNO_3_ solution, the blue solution undergoes a color change from blue to brown when exposed to UV light. In the case of Ag‐decorated HMB‐NTs, the presence of Ag NPs is clearly visible based on the prominent Ag NPs peak at a wavelength of 450 nm (Figure 1).

### 3.2. HRTEM Results

**FIGURE 2:**
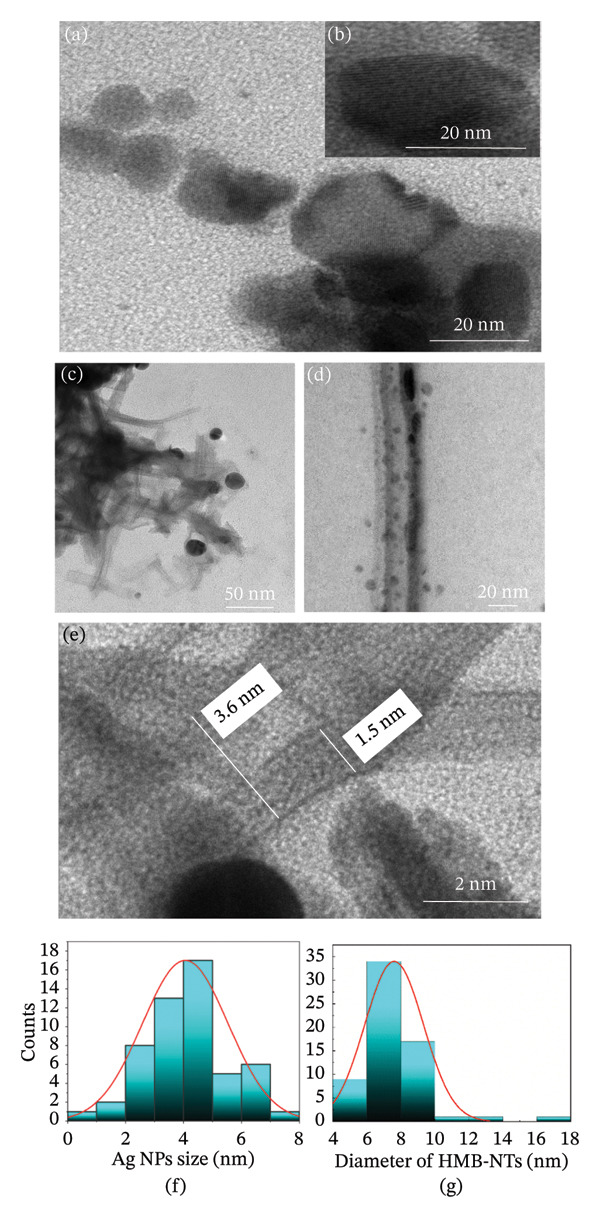
HRTEM images of (a) HMB‐NSHs, (b) single‐crystalline NSHs, (c) and (d) HMB‐NTs decorated with Ag NPs, and (e) the inside and outside diameters of the NTs measure approximately 1.5 and 3.6 nm, respectively. The dispersion of Ag NPs and the NT sizes are within the ranges of 3–5 nm and 6–10 nm, respectively, as demonstrated in panels (f) and (g).

As shown in Figure 2(a), NSHs exhibit a flat and extended two‐dimensional structure. The HMB‐NSH surfaces display a uniform distribution of nanoscale features. The lattice fringes observed in the HRTEM image (Figure 2(b)) confirm the crystalline nature of the NSHs. After adding AgNO_3_ solution into the HMB‐NSH solution, Ag cations (Ag^+^) accept electrons from Mo^+5^, leading to the rolling of HMB‐NSH and the generation of HMB‐NTs. As shown in Figures 2(c), 2(d), the NTs exhibit a tubular morphology with hollow interiors. Their inside and outside diameters are approximately 1.5 and 3.6 nm, respectively (Figure 2(e)). The HRTEM image (Figure 2(d)) further reveals the presence of Ag NPs decorating the surface of the NTs. An analysis of the histogram representing the size distribution of Ag NPs and the diameter of NTs reveals that the average size of Ag NPs (Figure 2(f)) is approximately 3–5 nm, whereas the diameter of the NTs averages around 6–10 nm (Figure 2(g)).

### 3.3. Chemical State and Crystal Structure

XPS measurements were carried out in order to precisely determine the chemical composition of the material’s surface, with particular focus on the Mo (3d) and O (1s) characteristic peaks of both HMB‐NSHs and Ag‐decorated HMB‐NTs. The Mo 3d core‐level peak evolution for the initial HMB‐NSHs and Ag‐decorated HMB‐NTs is shown in Figures 3(a), 3(b), respectively. In the case of HMB‐NSHs (Figure 3(a)), two peaks appear at 233.1 and 236.1 eV, corresponding to the 3d_3/2_ and 3d_5/2_ core‐level components, respectively. These peaks indicate a stoichiometric (Mo^+6^) oxide composition and 2 weak peaks at 231.9 and 234.9 eV related to Mo^+5^.

FIGURE 3High‐resolution XPS spectra of compounds HMB‐NSHs and HMB‐NTs, displaying the binding energies for Mo 3d and O 1s in panels (a–b) and (c–d), respectively. Furthermore, panel (e) exhibits the Ag 3d binding energy spectrum. Additionally, in panel (f), the XRD patterns of both the HMB‐NSHs (green trace) compound and HMB‐NTs (red trace) are shown.

**Figure:**
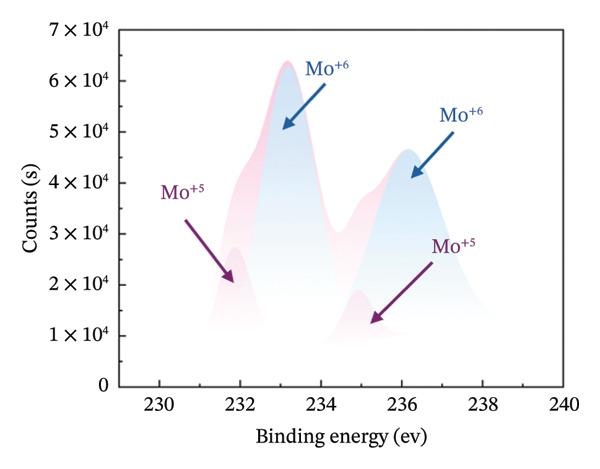
(a)

**Figure:**
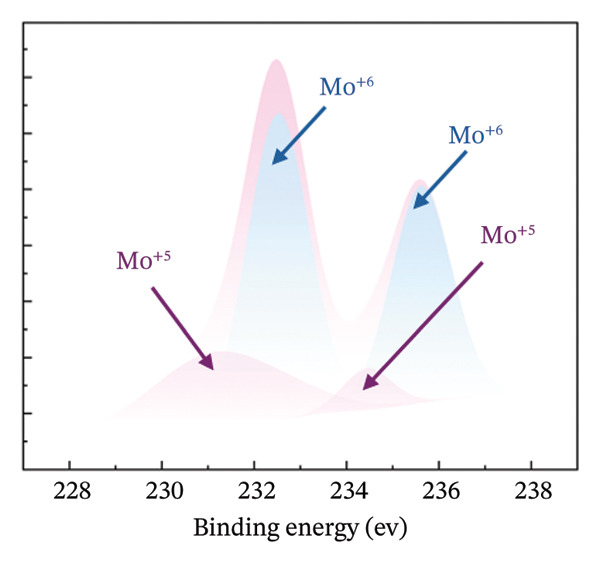
(b)

**Figure:**
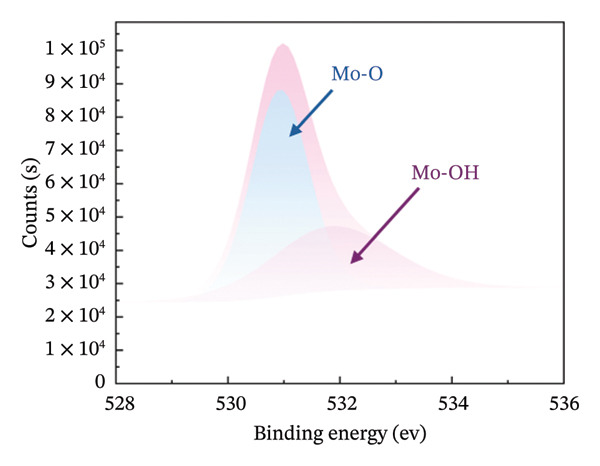
(c)

**Figure:**
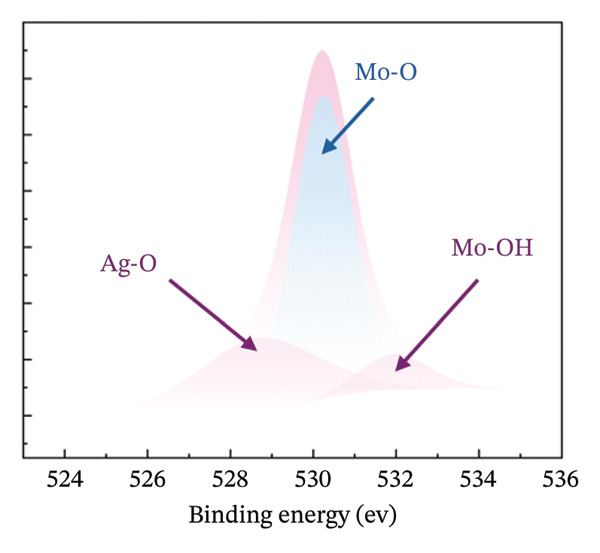
(d)

**Figure:**
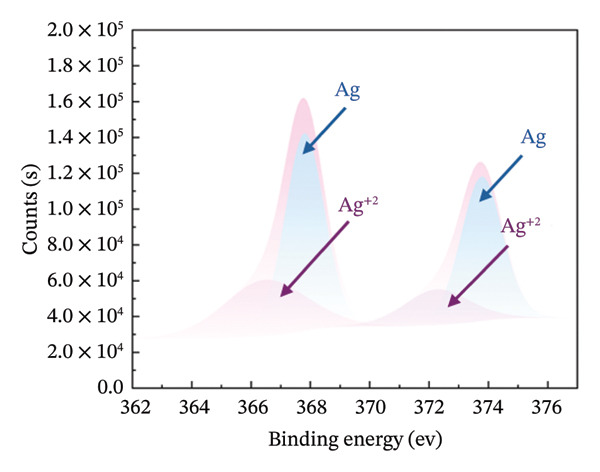
(e)

**Figure:**
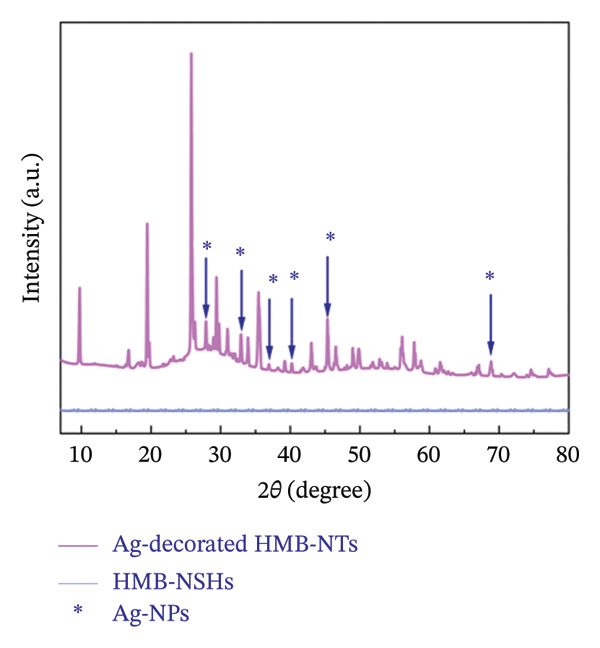
(f)

Similarly, for the Ag‐decorated HMB‐NTs, the binding energy of Mo is comparable to HMB‐NSHs, showing two distinct peaks at 232.5 and 235.7 eV, which can be attributed to MoO_3_ and Mo^+6^, respectively, and the two weak peaks at 231.3 and 234.5 eV are related to Mo^+5^ (Figure 3(b)). The XPS spectra for the O 1s core level, as illustrated in Figure 3(c), exhibit two peaks at 530.9 eV (Mo‐O) and 531.9 eV (Mo‐OH) for HMB‐NSHs and three characteristic peaks at 528.7 eV (Ag‐O), 530.2 eV (Mo‐O), and 532.0 eV (Mo‐OH) related to Ag‐decorated HMB‐NTs (Figure 3(d)). These peaks are associated with distinct oxygen environments: Ag‐O corresponds to oxygen atoms bonded to the metal Ag, Mo‐O is linked to Mo, and Mo‐OH arises from hydroxyl species adsorbed on the surface between Mo^+6^ (Figure 3(d)) [52, 53]. Notably, the O2 peak exhibits a sharper profile compared to the others. Figure 3(e) depicts the Ag 3d_5/2_ and Ag 3d_3/2_ peaks within the ranges of 365–369 eV and 372–375 eV, respectively. Within each peak, two distinct Ag oxidation states are identified; the Ag^0^ peaks are centered at 367.8 and 373.8 eV, while the Ag^+^ peaks are centered at 366.6 and 372.4 eV [54, 55]. The presence of the Ag_2_O layer on the surface of Ag NPs exposed to ambient oxygen leads to the appearance of the Ag^+^ peak (Figure 3(e)) [54, 56].

Figure 3(f) presents the XRD patterns of two compounds: HMB‐NSHs and Ag‐decorated HMB‐NTs. In the case of the HMB‐NSHs, initially presented as a blue suspension, the XRD pattern in Figure 3(f) (green trace) exhibits no discernible peaks. These observations support the idea that the lack of diffraction peaks can be ascribed to the thin nature of the colloidal species, as suggested by previous studies [57, 58]. Further clarification of this matter was achieved through HRTEM, EDS, and XPS analyses. For the compound Ag‐decorated HMB‐NTs, the XRD analysis reveals several peaks related to HMB‐NTs, notably seven distinct and sharp peaks at 2*θ* = 9.26°, 19.32°, 25.65°, 29.27°, 35.31°, 55.96°, and 57.69°. These peaks correspond to the (100), (200), (210), (111), (121), (212), and (331) planes of HMB‐NTs. However, the presence of Ag NPs in this compound results in the emergence of peaks at 2*θ* = 27.80°, 32.8°, 36.86°, 41.79°, 45.22°, and 68.56°, corresponding to the (110), (100), (200), (221), (410), and (521) planes of cubic Ag. All the diffraction peaks observed in the XRD pattern of HMB‐NTs (Figure 3(f) (red trace)) can be attributed to the crystal structure found in JCPDS 01‐083‐1173. The crystal size of Ag‐decorated HMB‐NTs is about 88.36 Å.

### 3.4. Mapping and EDS

**FIGURE 4:**
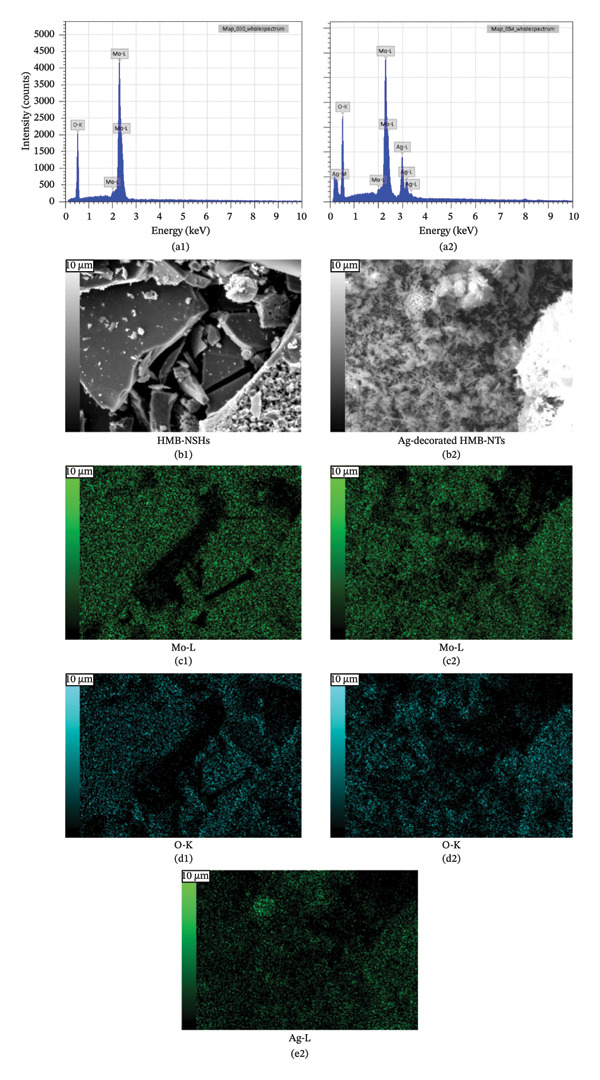
EDS analysis, elemental mapping of HMB‐NSHs, and Ag‐decorated HMB‐NTs. (a) and (b) depict the EDS spectra for HMB‐NSHs, and Ag‐decorated HMB‐NTs, confirming the presence of relevant elements. (e) and (g) show molybdenum and oxygen elements in HMB‐NSHs, examined at a 10‐micron scale (c, d) for both of them. Shifting to the Ag‐decorated HMB‐NTs sample, (f), (h), and (i) display molybdenum, oxygen, and Ag elements, respectively.

Figure 4 illustrates the SEM together with EDS microanalysis and elemental mapping of the pristine HMB‐NSHs and Ag‐decorated HMB‐NTs. The corresponding EDS survey spectra (Figures 4(a), 4(b)) confirm the elemental composition of the samples, showing characteristic signals of Mo (Mo–L) and O (O–K) for both materials. In the Ag‐decorated HMB‐NTs, an additional Ag signal is clearly detected (Figure 4(b)), evidencing successful Ag incorporation. It should be noted that hydrogen cannot be detected by EDS due to its very low atomic number; therefore, it is not expected to appear in the spectra or maps.

To clarify the spatial distribution of the elements, EDS elemental maps were acquired from the same SEM fields of view (Figures 4(c), 4(i)). Additionally, Figures 4(c) and 4(d) show the SEM images of the analyzed regions (scale bar: 10 μm), and the corresponding elemental maps were collected from the same positions for both samples. For HMB‐NSHs, the Mo and O maps (Figures 4(e), 4(g)) exhibit a uniform distribution across the analyzed area, indicating compositional homogeneity of the nanosheet framework. Similarly, for Ag‐decorated HMB‐NTs, the Mo and O signals remain broadly distributed throughout the sample (Figures 4(f), 4(h)), confirming preservation of the HMB matrix after decoration. Importantly, the Ag map (Figure 4(i)) reveals the presence and surface distribution of Ag within the same region, supporting that Ag is successfully introduced onto the HMB‐NTs rather than being absent or randomly contaminated. Overall, the combined EDS spectra and elemental maps validate the formation of Mo‐O frameworks in both samples and verify the incorporation of Ag in the Ag‐decorated HMB‐NTs. These results confirm the XRD and XPS analyses powerfully.

### 3.5. Antibacterial Analyses

#### 3.5.1. Planktonic Growth

As presented in Figure 5(a), all tested concentrations of HMB‐NSHs did not exhibit any antibacterial activity against the test organism. There is no difference in the growth patterns of cells inoculated with different concentrations of HMB‐NSHs, from 5 to 125 μg/mL, in comparison with the negative control (cells not exposed to any of the test agents), as the typical sigmoid bacterial growth curves were observed. However, the positive control (i.e., cells treated with Ciprofloxacin (125 μg/mL)—a standard and conventional antibacterial drug) showed a significant inhibition of planktonic growth of *B. subtilis.* It is important to emphasize that the concentrations employed for Ciprofloxacin have been maintained at 125 μg/mL. All the curves except for Ciprofloxacin (red trace) exhibited an initial lag phase (0–3 h), followed by an extended exponential growth phase (4–40 h), and a somewhat short death phase (42–48 h). The short decline observed at the 44th h and beyond could be conveniently attributed to exhaustion of nutrients and accumulation of toxic metabolic wastes in the growth medium, and definitely not to the HMB‐NSHs treatment. Our results indicate that HMBs have no intricate antibacterial activity against the test organism under the experimental conditions used. This agrees with the work of Akhidime et al. [59], where molybdenum achieved a limited antibacterial activity in all the methods used relative to zinc, copper, and Ag. This observation was attributed to the low leaching potential of molybdenum in the media. Although HMB is often used in various technological applications due to its electrical conductivity and catalytic activity [60–62]; its lack of certain functional groups in its chemical makeup that are necessary for interactions with bacterial cell membranes; its flat surface that prevents bacterial attachment; its inability to release harmful ions or electrons, which are necessary for disruption of enzymatic or biological processes through oxidative stress; and its stability under normal conditions that prevent it from undergoing chemical changes necessary to exhibit antibacterial activity may have contributed to its inactivity against bacteria as observed under the tested experimental conditions in the present study.

FIGURE 5Antibacterial effect of (a) HMB‐NSHs and (b) Ag‐decorated HMB‐NTs on planktonic growth of *B. subtilis* using optical density measurements (OD_600_). Data are presented as mean ± standard deviation from a minimum of three independent biological replicates.

**Figure:**
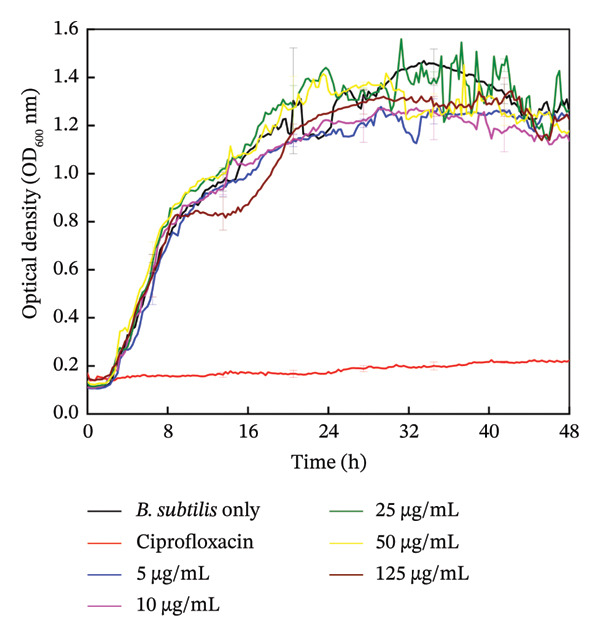
(a)

**Figure:**
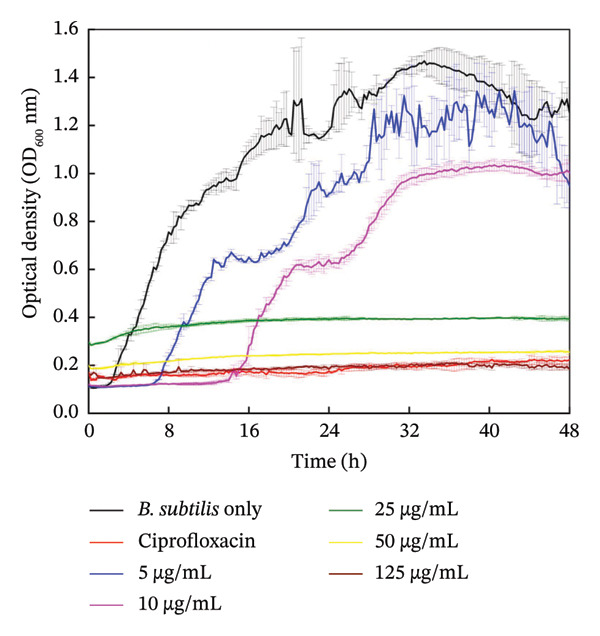
(b)

On the other hand, Ag‐decorated HMB‐NTs demonstrated a significant dose‐dependent antibacterial activity against *B. subtilis* (Figure 1(b)). This implies that as the concentration of Ag‐decorated HMB‐NTs increases, so does its capacity to inhibit bacterial growth. For context, at 32 h, which appears as the optimal time for planktonic growth of *B. subtilis* (Figure 5), 5 μg/mL Ag‐decorated HMB‐NTs caused approximately 17.4% reduction in planktonic growth, while 10 μg/mL Ag‐decorated HMB‐NTs achieved about 31.2% reduction. Interestingly, 25 μg/mL Ag‐decorated HMB‐NTs demonstrated a significant reduction of about 75.7%, while at 50 μg/mL Ag‐decorated HMB‐NTs and beyond, no increase in optical density readings was observed, indicating that planktonic growth has been completely inhibited at these concentrations. Such dose‐dependent responses are critical in clinical practice because they offer important information about the ideal treatment concentrations needed to effectively control specific bacterial strains [63]. In practical terms, the ability of Ag‐decorated HMB‐NTs to significantly inhibit the proliferation of planktonic cells of *B. subtilis* is indicative of its potential ability to prevent the initial onset of bacterial infection. This observation highlights both the importance of NPs design and the potential of specially designed NPs to inhibit planktonic bacterial growth, which is essential for delaying the onset of many diseases.

#### 3.5.2. Biofilm Growth

Figures 6 and 7 are the crystal violet estimates of biofilm formation and removal of *B. subtilis* under the treatment of different concentrations of HMB‐NSHs and Ag‐decorated HMB‐NTs. Following the graphical assessment, we observed that HMB‐NSHs exhibited no substantial activity against biofilm formation (Figure 6(a)) and removal (Figure 7(a)) of *B. subtilis*, even at concentrations as high as 1000 μg/mL HMB‐NSHs. This is not surprising as it is consistent with our observation when applied against planktonic cells (Figure 5(a)). Statistical analysis, however, indicates that in biofilm formation experiments, a significant difference exists between the untreated cells (negative control) and the cells treated with 5, 10, and 25 μg/mL HMB‐NSHs, while no statistical significance exists at higher concentrations (Figure 6(a)). In the case of biofilm removal, none of the concentrations tested showed a significant difference in comparison with the untreated cell (Figure 7(a)). In contrast, Ag‐decorated HMB‐NTs demonstrated a strong dose‐dependent activity against biofilm formation (Figure 6(b)) of *B. subtilis* and disruption of those already formed 48 h before the addition of the test agent (Figure 7(b)). Statistical analysis shows that, for biofilm formation, lower concentrations of 5 and 10 μg/mL exhibited no significant difference in comparison with the untreated cells. Interestingly, 25 μg/mL Ag‐decorated HMB‐NTs achieved a significant biofilm inhibition of approximately 77.7%, while higher concentrations of 50 and 125 μg/achieved more than 96% inhibition (Figure 7(b)).

FIGURE 6Effect of: (a) HMB‐NSHs and (b) Ag‐decorated HMB‐NTs on biofilm formation of *B. subtilis* via crystal violet staining assay. Data are presented as mean ± standard deviation from a minimum of three independent biological replicates. NS means not significant, The symbol “∗” means statistically different from untreated *B. subtilis* cells (negative control), The symbol “∗∗” means statistically significant from untreated *B. subtilis* cells but shows insignificant difference in comparison to the positive control (Ciprofloxacin) according to ANOVA followed by Tukey’s test at *p* < 0.05.

**Figure:**
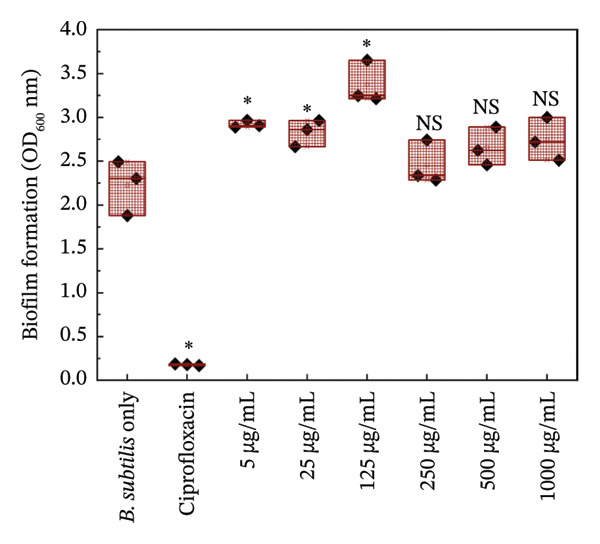
(a)

**Figure:**
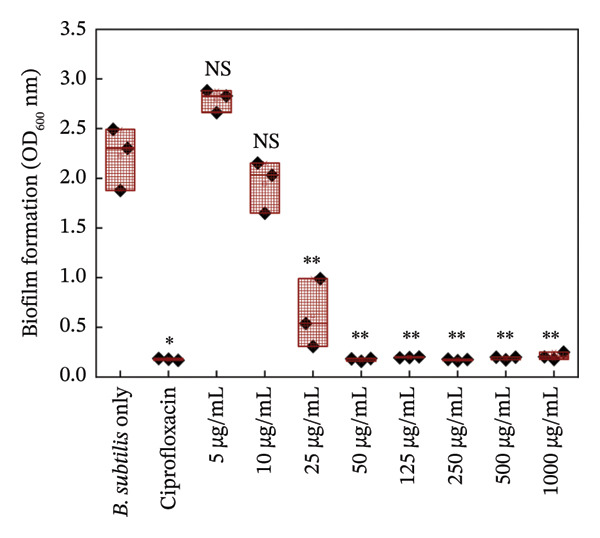
(b)

FIGURE 7Effect of: (a) HMB‐NSHs, and (b) Ag‐decorated HMB‐NTs on already formed biofilm of *B. subtilis* (biofilm removal estimates) via crystal violet staining assay. Data are presented as mean ± standard deviation from a minimum of three independent biological replicates.

**Figure:**
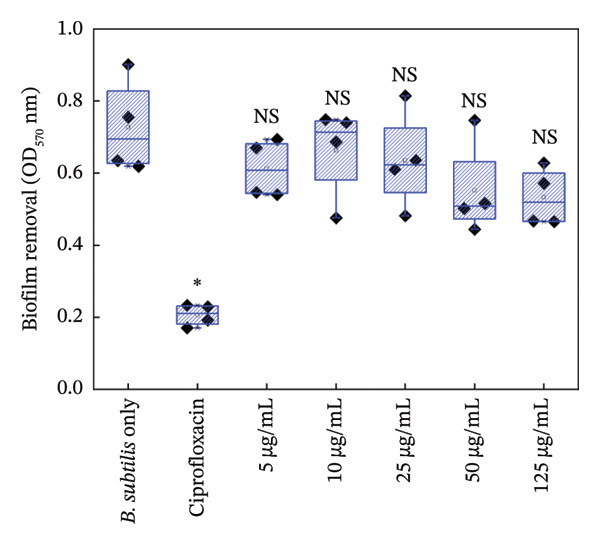
(a)

**Figure:**
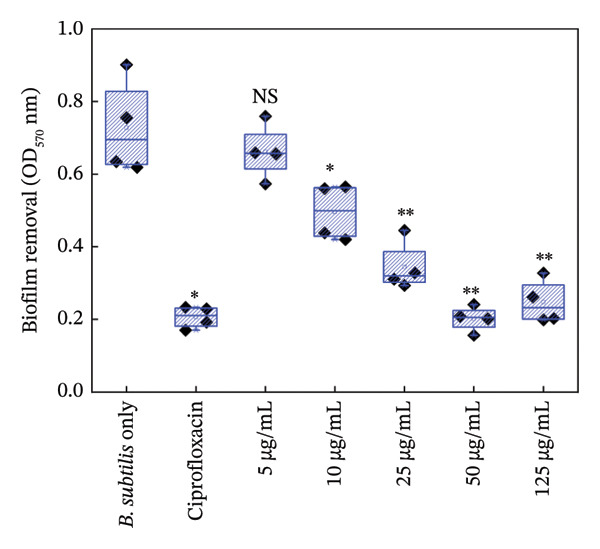
(b)

Further insight into the statistical analyses also shows that 50 μg/mL Ag‐decorated HMB‐NTs performed as competitively as possible as the conventional antibacterial drug (125 μg/mL Ciprofloxacin), as both test concentrations achieved approximately more than 96% biofilm inhibition. Results for biofilm removal, on the other hand, follow a similar pattern to that of biofilm formation. For context, at lower concentrations of 5 μg/mL Ag‐decorated HMB‐NTs, no significant antibiofilm activity was observed. However, at 10, 25, and 50 μg/mL, significant 35.7%, 64.3%, and 72.9% biofilm removals, respectively, were achieved. Similarly, 50 μg/mL Ag‐decorated HMB‐NTs demonstrated a competitive performance as 125 μg/mL Ciprofloxacin—a conventional antibacterial agent, as both test agents achieved approximately more than 70% removal of already formed biofilms of *B. subtilis* (Figure 7(b))*.* The ability of Ag‐decorated HMB‐NTs to demonstrate both inhibition and removal efficacy against *B. subtilis* biofilms indicates a multifaceted mechanism of action, making it a good candidate for further exploration and development in the field of antibiofilm agents. The difference in the activities of HMB‐NSHs and Ag‐decorated HMB‐NTs could be explained by the extra Ag metal conjugated on the molybdenum hydrogen complex, a phenomenon similar to our recently published findings [64]. Our results in the current study are suggestive of an improved antibacterial activity through synergistic mechanisms between Ag and HMB, thereby making the conjugated form a potent antibacterial agent with antiplanktonic and antibiofilm efficacy.

#### 3.5.3. Spectroscopic Analysis

**FIGURE 8:**
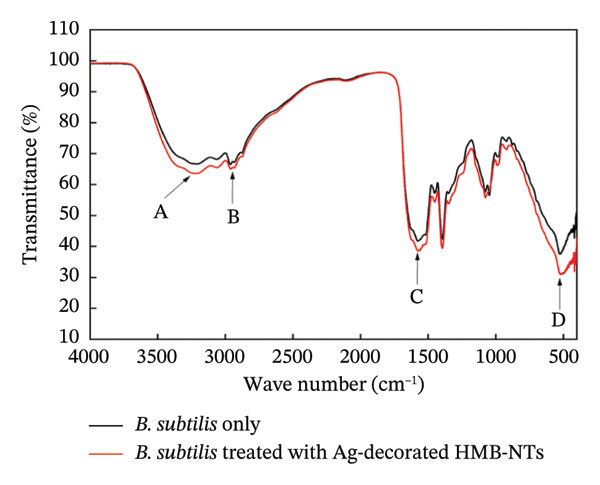
Representative FTIR spectra of *B. subtilis* cells only (negative control—black trace) and *B. subtilis* cells treated with 50 μg/mL Ag‐decorated HMB‐NTs (red trace).

FTIR analysis was carried out by comparing the peaks between the untreated *B. subtilis* cells (negative control) and bacterial cells treated with Ag‐decorated HMB‐NTs. Prior to the analysis, all unbound organic components including media and NPs were washed off from the samples, leaving only cellular materials. As presented in Figure 8, the spectrum of the treated cells was evidently different from that of the untreated cells. In general, the O‐H and N‐H stretching that correspond to polysaccharides and proteins were observed at 3410 cm^−1^ (A); C=O stretching corresponding to polypeptide and protein backbone was observed at 1570 cm^−1^ (C), while carbon moieties corresponding to the glycogen were observed at 580 cm^−1^ (D). Meanwhile, the spectrum of Ag‐decorated HMB‐NTs‐treated bacterial cells also shows a similar spectrum but with a reduction in all observed peaks, indicating surface interactions and eventual antibacterial action. Overall, FTIR spectra indicate the involvement of polysaccharides and polypeptides of the bacterial cell wall in surface interaction with the Ag‐decorated HMB‐NTs on the bacterial cell surface. The use of FTIR spectra of cellular constituents has been used to investigate minor or significant changes in cellular composition caused by chemical and/or biological therapies. In previous studies [65, 66], microbiological examinations in conjunction with FTIR spectroscopy have also demonstrated connections between bacterial viability and the presence of comparable functional groups to those mentioned in the current study.

#### 3.5.4. Microscopic Analyses

Figure 9 is a SEM image of *B. subtilis* cells with and without treatment. At × 1,00,000 magnification, the untreated bacterial cells exhibit a fully formed biofilm consisting of moderately elongated cells (with intact structural integrity) enclosed in a polymeric matrix (Figure 9(a)). Moreover, *B. subtilis* treated with HMB‐NSHs demonstrated a somewhat well‐formed biofilm but with less pronounced extracellular polymeric matrix coupled with the formation of somewhat wrinkled membrane surfaces (Figure 9(b)). This is suggestive that the bacterial cells recognize HMB‐NSHs as an extraneous molecule but with a significantly less antibacterial efficacy. In contrast, Ag‐decorated HMB‐NTs significantly reduced the biofilm biomass, while the few surviving cells showed morphological distortions and pronounced rough surfaces (Figure 9(c)). No visible bacterial cells were observed when Ciprofloxacin was applied. Instead, a mass of cellular debris resulting from the biocidal effect was seen (Figure 9(d)).

FIGURE 9Representative scanning electron micrographs of *B. subtilis cells* (a) not treated with any of the tested compounds; (b) treated with HMB‐NSHs; (c) treated with Ag‐decorated HMB‐NTs; and (d) treated with Ciprofloxacin.

**Figure:**
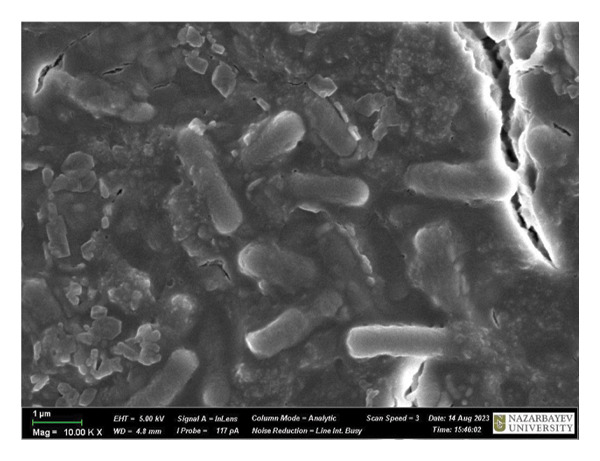
(a)

**Figure:**
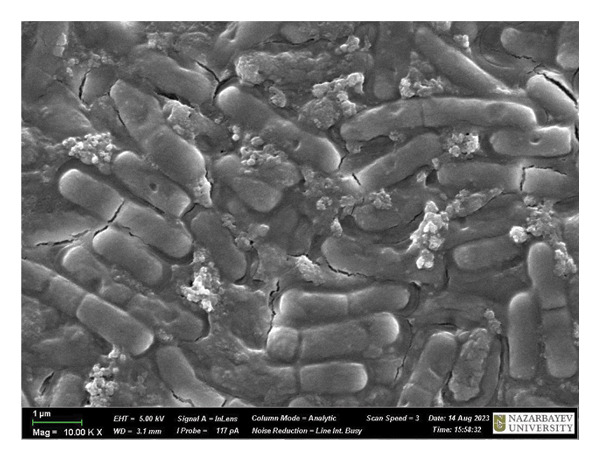
(b)

**Figure:**
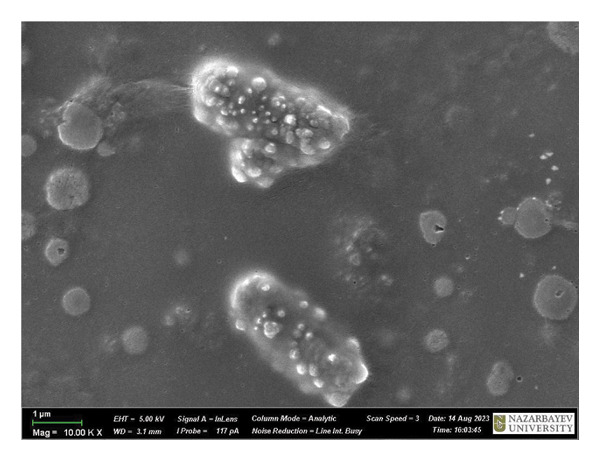
(c)

**Figure:**
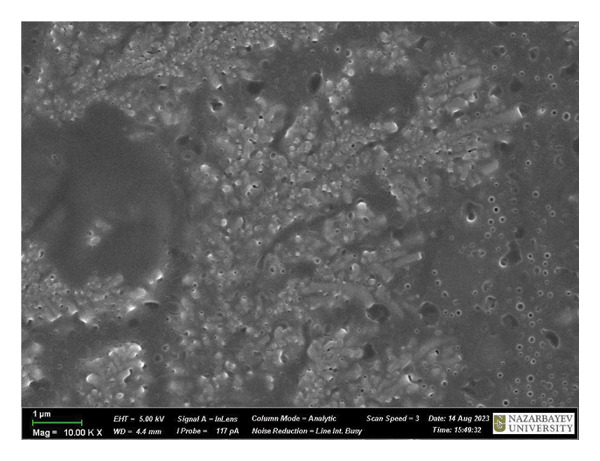
(d)

The activity of Ag‐decorated HMB‐NTs could be explained through interaction between the negatively charged peptidoglycan cell surface in *B. subtilis* and the positively charged Ag‐decorated HMB‐NTs particles, potentially leading to the deposition of cytotoxic ions in the cell [15, 67, 68]. The antibacterial effect of Ag‐decorated HMB‐NTs may have been initiated following the attachment of molybdenum and Ag ions to the bacterial cell wall. As previously proposed, NP ions could be released when the NPs were mixed with the culture medium even before attachment. In the case of HMB‐NSHs, they demonstrated strength to significantly eradicate *B. subtilis* when decorated with Ag NPs and undergoing a change to NTs morphology, which intensifies its antibacterial efficacy. Subsequent interactions may have involved the binding of metallic Ag NPs and Mo^+^ to the proteins and other macromolecules, leading to disruption of their intramolecular bonds [69]. Also, the strong oxidizing potential of Ag–HMB‐NTs in conjunction with its efficient reactive oxygen species (ROS) production may have triggered a series of physiological changes, including membrane disintegration, malfunctioning of cellular proteins, and the inhibition of DNA replication [67, 70–72].

Furthermore, silver nanomaterials, as previously mentioned, are well‐established as potent antimicrobials; however, they also exhibit significant cytotoxic effects toward mammalian cells [73, 74] In vitro studies have shown that Ag NPs are toxic to numerous human cell lines (including lung epithelial cells, endothelial cells, red blood cells, keratinocytes, and liver cells), with the toxicity generally dose‐, size‐, and time‐dependent. Smaller particles (≲ 10 nm) tend to be more cytotoxic due to easier cellular uptake and dissolution, and Ag NP exposure can even allow particles or ions to cross biological barriers (e.g., the blood–brain barrier) and accumulate in organs [75, 76]. Mechanistically, silver nanomaterials induce oxidative stress and cell damage through multiple pathways. Intact Ag NPs can directly interact with cell membranes to trigger lipid peroxidation and protein misfolding, leading predominantly to proteotoxic stress and necrotic cell death, whereas released Ag^+^ ions from the particles increase ROS inside cells and tend to induce apoptotic pathways. This combination of nanoparticle and ionic mechanisms means Ag‐based nanomaterials act as a double‐edged sword: They effectively kill microbes but can also harm host cells in higher organisms [77, 78]. In vivo studies on animals further show that Ag NPs can distribute and accumulate in the liver, spleen, kidneys, lungs, and brain following exposure, highlighting their potential for systemic toxicity with prolonged or high‐dose use [79–83]. Taken together, the known cytotoxic profile of Ag nanomaterials involves broad‐spectrum antimicrobial action coupled with a risk of collateral cytotoxicity to eukaryotic cells.

Given the concerns with free Ag NPs, the HMB‐NTs are designed to mitigate those issues. The expected behavior of HMB‐NTs is that the NT “container” will moderate the release of the active Ag to the surroundings, thereby reducing acute toxicity while retaining antimicrobial efficacy [84–86]. This means that, instead of a sudden burst of Ag ions (which could kill human cells along with bacteria), the HMB‐NT system would gradually and slowly diffuse the toxic Ag ions. Indeed, studies on Ag‐loaded HNTs have shown that the NTs significantly delay and prolong the release of Ag ions, achieving sustained antibacterial action over several days with minimal initial spike in concentration [87]. By preventing sharp exposure peaks, this slow‐release behavior would minimize the cytotoxic impact on eukaryotic cells (since cell cultures can better tolerate lower, sustained doses than a high acute dose). Overall, we hypothesize that HMB‐NTs will exhibit targeted antimicrobial effect with a more benign profile in biological systems compared to free nanoparticles. This hypothesis is supported by the known biochemical behavior of nanocarriers, which have demonstrated high biocompatibility even after surface modifications [88–90]. In summary, HMB‐NTs would maintain potent activity against microbes as revealed in the current study while significantly dampening the cytotoxic side effects through controlled release and the biocompatible nature of their NT matrix. Consequently, given the complexity of mammalian cells and their distinct responses to nanomaterials, future studies in our laboratory will focus on detailed cytotoxicity profiling in relevant cultured cell models. These investigations will be essential to ensure that HMB‐NTs, as a promising and adaptable nanomaterial platform, meet the necessary safety benchmarks for translational biomedical applications.

Additionally, the fluorescence micrographs (Figure 10) complemented the SEM results and further revealed the degree and dynamics of biofilm formation in both the presence and absence of the antibacterial treatment. The varying intensities captured in these images offer a nuanced perspective on the efficacy of the test agents against biofilm growth of *B. subtilis*. Figure 10(a) shows the negative control (untreated cells), which showed the highest fluorescence, suggesting that there is no hindrance to bacterial adhesion or biofilm formation/maturation processes, allowing the biofilm to form undisturbed. This is almost similar to fluorescence observed in the presence of HMB‐NSHs, indicating that this compound has little to no effect on biofilm development (Figure 10(b)). However, in the presence of Ag‐decorated HMB‐NTs, a significantly weak fluorescence signal was obtained, indicating a reduction in biofilm density, potentially demonstrating the successful disruption of bacterial adhesion or the eradication of mature biofilm structures. The faded intensity suggests that Ag‐decorated HMB‐NTs treatment might have disrupted certain biofilm‐associated physiological processes as previously explained, allowing partial biofilm development to occur (Figure 10(c)). Lastly, Ciprofloxacin‐treated cells showed no intensity, indicating its potent inhibitory effect on biofilm formation (Figure 10(d)). The combined efficiency of Ag and molybdenum ions may have been responsible for the observed inhibitory effects on planktonic growth, biofilm formation, and biofilm removal of *B. subtilis*. These ions can adhere to the cell membrane of microorganisms, disrupt the integrity and permeability of the cell membrane, prevent the transport and exchange of biomolecules, or cause leakage of intracellular constituents [70, 72, 91]. Following the penetrative interaction, they may disrupt the respiratory enzymes or disrupt the electron transfer chain, which would inhibit cellular respiration and cause the generation, release, and build‐up of toxic ROS [67, 92]. Overall, the mechanism of action of NPs is complex and could be facilitated by a number of factors or conditions [93].

FIGURE 10Representative fluorescence electron micrographs of *B. subtilis cells* (a) not treated with any of the tested compounds; (b) treated with HMB‐NSHs; (c) treated with Ag‐decorated HMB‐NTs; and (d) treated with Ciprofloxacin.

**Figure:**
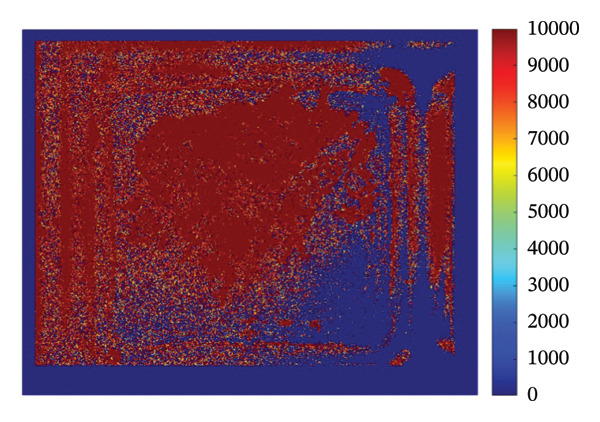
(a)

**Figure:**
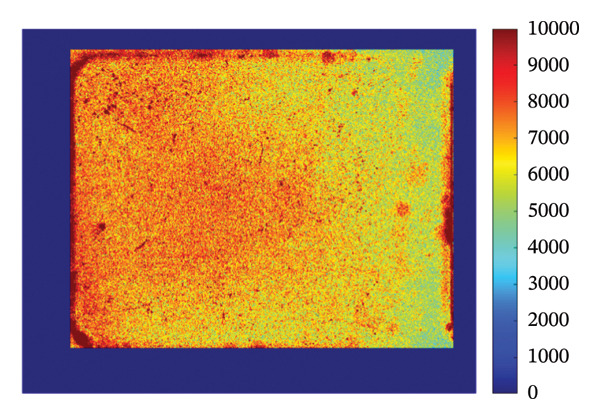
(b)

**Figure:**
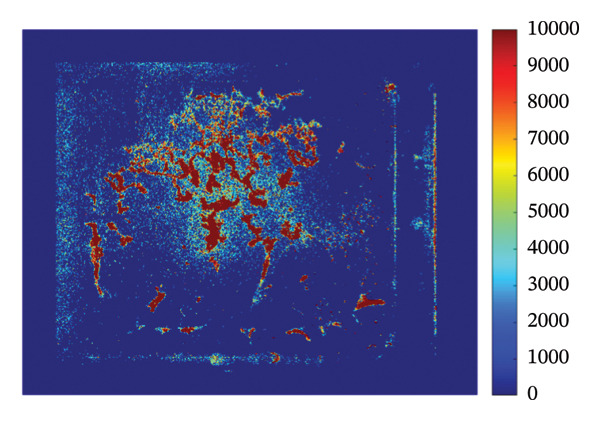
(c)

**Figure:**
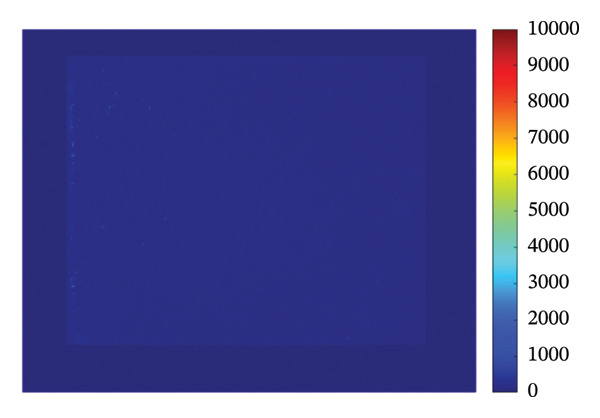
(d)

### 3.6. Computational Outputs

Two distinct protein structures from *Escherichia coli*, identified by PDB codes 4WO7 and 2VAM, were selected to investigate the potential antibacterial mechanisms of specific ligands. The 4WO7 protein is a component of the bacterial cell wall [94], making it an ideal target to evaluate whether the ligands can bind to and disrupt this structural barrier, potentially inducing cell lysis or death. In contrast, 2VAM is an intracellular protein involved in the bacterial cell cycle [95]. This dual selection was deliberate, enabling the examination of two distinct scenarios: (1) direct cell‐wall disruption and (2) the ligands’ potential effects after crossing the cell wall, whether through passive diffusion, transport systems, or receptor‐mediated uptake to interact with critical intracellular targets. This strategy allows for a more comprehensive assessment of the compounds’ antibacterial potential, accounting for both extracellular and intracellular modes of action.

Molecular docking results (Figure 11) demonstrate that both Ag and HMB form significant interactions with the active sites of 4WO7 and 2VAM. However, it is important to acknowledge the inherent limitations of AutoDock4, particularly in handling metal‐containing complexes, as well as the constraints of LigPlot+ [41] for 2D visualization. While these tools are effective for standard organic ligand–protein interactions, they often inadequately represent coordination bonds involving transition metals or metalloid species. To address these limitations and obtain a more detailed electronic‐level understanding of the ligand–protein interactions, we employed DFT. This quantum mechanical approach enables a deeper investigation of binding geometries, energy profiles, and electron density redistribution. Using DFT, we achieved an atomistic‐resolution view of the molecular interactions, offering further insights into the binding behavior and potential bioactivity of Ag and HMB with their protein targets.

FIGURE 11Three‐dimensional representation of docking results showing interactions of (a, c) Ag and (b, d) HMB with (right panel) the cell‐wall protein (PDB ID: 4WO7) and (left panel) the intracellular division regulator protein (PDB ID: 2VAM) of *Bacillus subtilis*. Panels (a) and (c) display Ag–protein interactions, and panels (b) and (d) show HMB–protein interactions.

**Figure:**
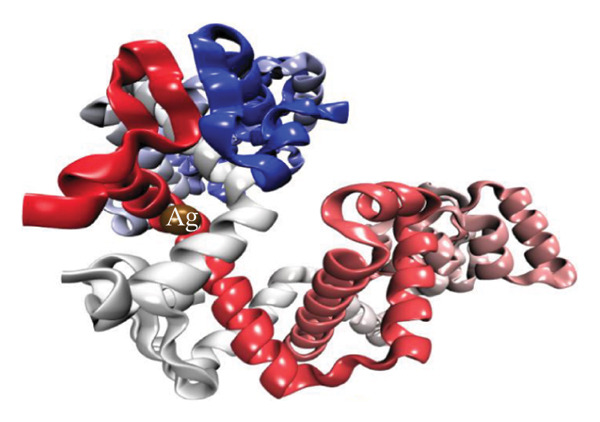
(a)

**Figure:**
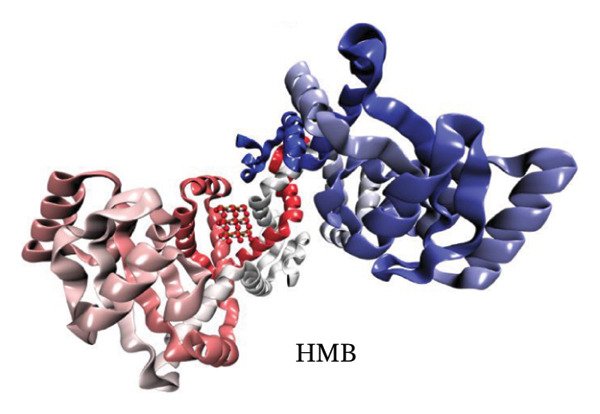
(b)

**Figure:**
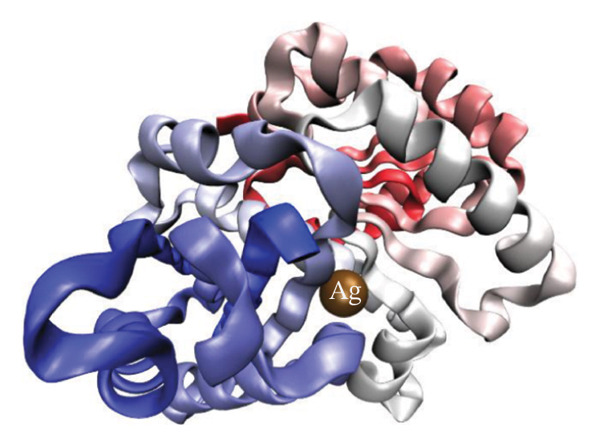
(c)

**Figure:**
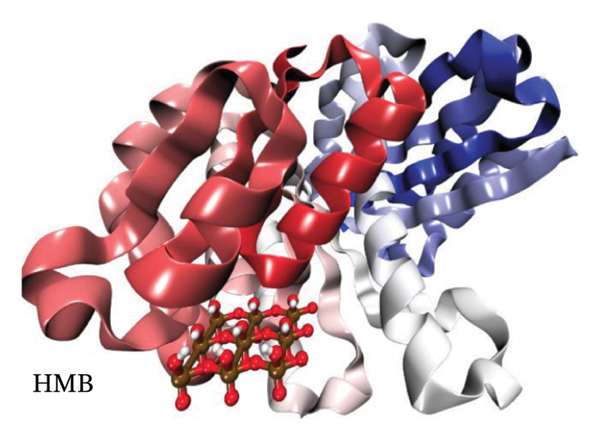
(d)

The ligand–protein configurations obtained from molecular docking calculations were prepared for DFT calculations using LigPlot + software. Single‐point energy calculations were performed using the B3LYP/6‐311++G(d,p) method to obtain wavefunctions for further analysis. The calculated wavefunctions were then used to compute QTAIM topological indices, including electron density (*ρ*(r)), Laplacian of electron density (∇^2^ *ρ*(*r*)), and ellipticity index (*ε*), at all possible bond critical points (BCPs) between Ag/HMB and the proteins.

A BCP, which indicates possible weak or strong interactions between atoms, is a critical point (where ∇*ρ*(*r*) = 0) with two negative eigenvalues (*λ*) of the Hessian matrix of electron density (∇^2^ *ρ*(*r*)) and one positive eigenvalue. The values of *ρ*(*r*) and ∇^2^ *ρ*(*r*) at BCPs can classify interaction types. As a general guideline:

• *ρ*(*r*) > 0.2 with negative ∇^2^ *ρ*(*r*) indicates strong covalent interactions with electron accumulation between nuclei
• *ρ*(*r*) ≤ 0.1 with positive ∇^2^ *ρ*(*r*) suggests ionic interactions between cations and anions
• 0.01 ≤ *ρ*(*r*) ≤ 0.04 with slightly positive ∇^2^ *ρ*(*r*) characterizes hydrogen bonding
• *ρ*(*r*) ≤ 0.01 a.u. with small or slightly negative ∇^2^ *ρ*(*r*) indicates weak vdW interactions

The bond ellipticity (*ε*) helps distinguish bonding regimes. In QTAIM:

• *ε* = 0 indicates cylindrically symmetric σ‐bonds
• Increasing *ε* values reflect greater bond anisotropy and π‐character
• vdW interactions typically show *ε* ≈ 0, with increasing *ε* values corresponding to decreased interaction stability
• *ε* ≈ 0 suggests more electrostatic, noncovalent interactions (NCI)

Figure 12 presents QTAIM analysis results for Ag and HMB interactions with both proteins, while Table S1 (see Supporting information) reports the *ρ*(*r*), ∇^2^ *ρ*(*r*), and *ε* values for the most significant BCPs. RDG analysis results appear in Figure S1 (Supporting information), and IGM analysis results in Figure S2 (Supporting information).

FIGURE 12QTAIM analysis of interactions between (top panel) Ag atoms and (bottom panel) HMB NPs with amino acids from *Bacillus subtilis* proteins (a, b: PDB ID 4WO7; c, d: PDB ID 2VAM). Atom color scheme: H (white), C (gray), N (blue), O (red), Ag (brown), Mo (ochre). Bond critical points are indicated by violet spheres, with bond paths shown as cyan lines.

**Figure:**
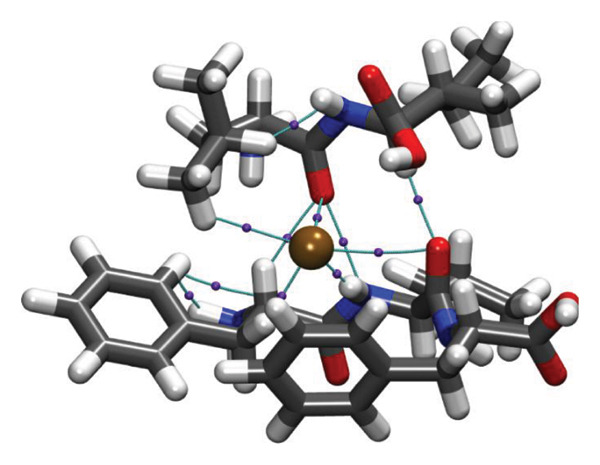
(a)

**Figure:**
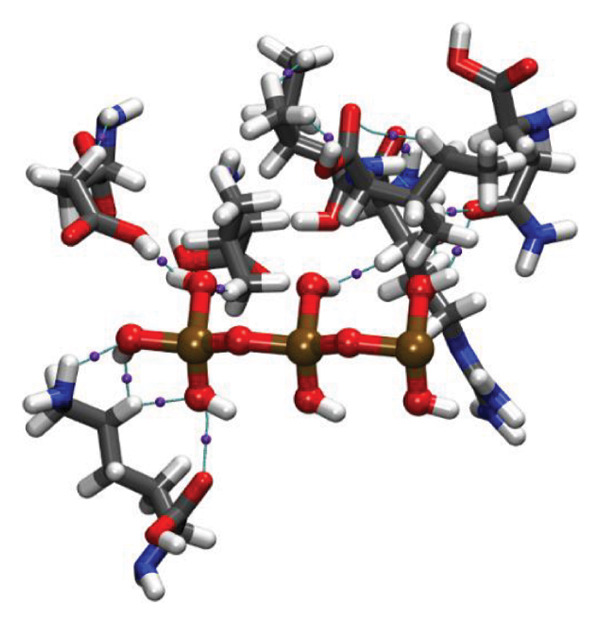
(b)

**Figure:**
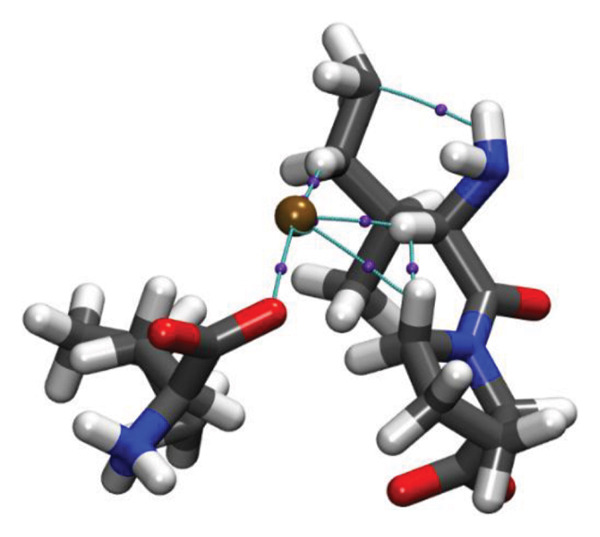
(c)

**Figure:**
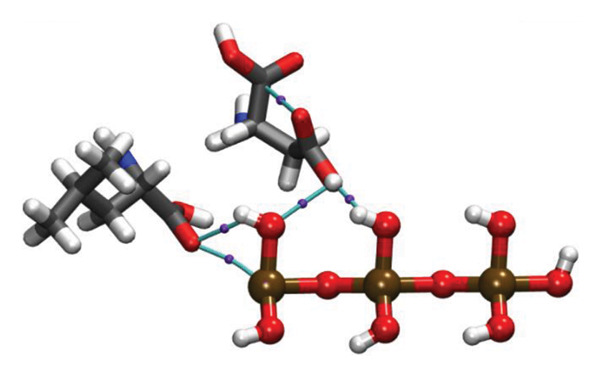
(d)

#### 3.6.1. Antibacterial Mechanism via Cell‐Wall Disruption

The dominant interactions in the Ag–4WO7 complex primarily involve coordination between the Ag atom and specific amino acid residues: a relatively strong interaction with valine (Val, *ρ*(*r*) = 0.039 a.u.) and weaker interactions with tyrosine (Tyr) and leucine (Leu, *ρ*(*r*) ≈ 0.02 a.u.). These interactions manifest as characteristic peaks in the RDG plots. Analysis of HMB‐4WO7 interactions reveals stable hydrogen bonding networks, as evidenced by electron densities (0.01 ≤ *ρ*(*r*) ≤ 0.03 a.u.), positive Laplacian values (∇^2^ *ρ*(*r*) > 0), and minimal ellipticity (*ε*). The most significant hydrogen bonds form between HMB’s hydroxyl group and glutamine (Gln, *ρ*(*r*) ≈ 0.032 a.u.), and between HMB and lysine (Lys)/valine (Val) residues (*ρ*(*r*) ≈ 0.023 a.u.), corresponding to distinct features in the sign(*λ*_2_)*ρ*(*r*) plot at −0.032 and −0.023 a.u., respectively.

Complementary IGM analysis (Figure S2) confirms substantial NCIs between both Ag/HMB and the 4WO7 protein (Figures S2(a), S2(b)). The extensive green isosurfaces indicate strong attractive interactions (hydrogen bonds and vdW forces), where the surface volume directly correlates with interaction strength. These robust interfacial interactions demonstrate NPs’ ability to penetrate and bind to bacterial surface proteins, providing a mechanistic basis for their observed antibacterial activity against *Bacillus* species. The combined QTAIM, RDG, and IGM results consistently show that both Ag (Figures S2(a), S2(c)) and HMB (Figures S2(b), S2(d)) form stable interactions with critical cell‐wall components, disrupting bacterial membrane integrity and function, which are consistent with the experimental findings.

#### 3.6.2. Alternative Antibacterial Mechanism via Intracellular Disruption

An alternative antibacterial mechanism can be proposed, in which the constituent particles of the synthesized nanoparticle (Ag and HMB) penetrate bacterial cells and disrupt intracellular metabolic processes. To investigate this possibility, we examined interactions with the 2VAM protein, which plays a direct role in cell cycle regulation and division. Following the same methodology as our previous analyses, we focus here on key results.

QTAIM data (Table S1 in Supporting information) reveal that interactions with 2VAM are generally weaker than those with 4WO7, as evidenced by lower electron density values (*ρ*(*r*)). All Ag–2VAM interactions are stable but weak vdW type, characterized by low *ρ*(*r*) (0.012–0.02 a.u.), positive ∇^2^ *ρ*(*r*), and small ellipticity (*ε*) indices. The strongest interaction occurs between Ag and isoleucine (Ile) residues, visible as a distinct peak at sign(*λ*_2_)*ρ* ≈ −0.021 in RDG plots (Figure S1). The negative *λ*_2_ value confirms the attractive nature of this interaction, which stabilizes the Ag–protein complex. Figure S1 shows NCI analysis through RDG isosurface plots for Figures S1(a), S1(c) Ag and Figures S1(b), S1(d) HMB complexes with *Bacillus subtilis* proteins (a, b: 4WO7; c, d: 2VAM).

HMB forms hydrogen bonds with 2VAM (0.01 ≤ *ρ*(*r*) ≤ 0.025 a.u., ∇^2^ *ρ*(*r*) > 0), though these are weaker than its interactions with 4WO7. Higher *ε* values indicate greater bond anisotropy and reduced stability, suggesting that these interactions are more easily disrupted. The primary HMB–2VAM interaction involves hydrogen bonding with Ile, appearing as a feature near −0.021 in the RDG plot (Figure S1(d)). Comparative analysis of RDG plots reveals that NPs–4WO7 (Figures S1(a), S1(b)) interactions are predominantly attractive, with minimal density in repulsive regions (sign(*λ*_2_)*ρ*(*r*) > 0). In contrast, 2VAM complexes (Figures S2(c), S2(d)) show clear repulsive components, confirming weaker binding. IGM analysis (Figure S2) further supports these findings: the smaller green isosurfaces for 2VAM complexes indicate less extensive vdW interactions compared to 4WO7. While attractive forces still exist between NPs and 2VAM, their reduced intensity explains the lower binding affinity. Figure S2 shows IGM isosurface analysis of NCIs between Figures S2(a), S2(c) Ag and Figures S2(b), S2(d) HMB ligands with *Bacillus subtilis* cell‐wall proteins (a, b: 4WO7; c, d: 2VAM). Green isosurfaces indicate weak attractive vdW interactions, where surface extent correlates with interaction strength. Isosurfaces were generated at δg = 0.03 a.u. with a promolecular density approximation, using *a* ±0.05 a.u. density cutoff for visualization. The isosurface value was set to 0.5 a.u. for optimal visualization of intermolecular interactions.

Based on these results, we propose a dual antibacterial mechanism. First, HMB facilitates initial attachment to the cell wall (4WO7) via strong hydrogen bonds, while Ag interacts strongly with surface proteins (*ρ*(*r*) up to 0.039 a.u.), compromising membrane integrity. Second, dissolved Ag^+^ ions penetrate the cell and bind to intracellular targets like 2VAM. Although these interactions are weaker (*ρ*(*r*) ≈ 0.02 a.u.), they are sufficient to disrupt cell cycle regulation. The combined effects of membrane destabilization and metabolic interference explain the NPs’ potent antibacterial activity against *B. subtilis*.

## 4. Conclusions

The successful synthesis and multiscale characterization of two novel nanostructures, HMB‐NSHs and Ag‐decorated HMB‐NTs, have been demonstrated. Structural elucidation through HRTEM, XPS, EDS, and elemental mapping confirmed the formation of crystalline, ultrathin HMB scaffolds, and the uniform deposition of Ag nanoparticles (∼3–5 nm) on NTs with diameters of ∼1.5–3.6 nm. FTIR spectra revealed the depletion of characteristic O–H, N–H, and C=O stretching bands following treatment, indicating direct interaction of Ag‐decorated HMB‐NTs with polysaccharides and proteins on the bacterial cell surface. While HMB‐NSHs alone were biologically inert, functionalized Ag‐decorated HMB‐NTs exhibited potent antibacterial activity against *Bacillus* subtilis, significantly inhibiting both planktonic growth and biofilm development. Spectroscopic and microscopic analyses revealed that Ag‐decorated HMB‐NTs interact directly with bacterial cell‐wall constituents, disrupting membrane integrity and inducing oxidative stress. Computational investigations further confirmed dual‐site binding of Ag and HMB to both extracellular (PBP, PDB ID: 4WO7) and intracellular (FtsZ, PDB ID: 2VAM) protein targets, establishing a multistage antibacterial mechanism. Collectively, these findings validate that Ag‐decorated HMB‐NTs act as a dual‐function nanomaterial: The HMB scaffold promotes surface adhesion and stability, while Ag facilitates membrane damage and protein interference, highlighting them as next‐generation antibacterial candidates, particularly against biofilm‐forming bacteria. Future studies should focus on evaluating long‐term cytotoxicity, environmental impact, and therapeutic synergy with conventional antibiotics. Overall, Ag‐decorated HMB‐NTs represent a significant advancement in the development of multifunctional, silver‐based nanomaterials for combating biofilm‐associated bacterial threats across biomedical and biotechnological domains.

## Funding

This work was supported by Kazakhstan Ministry of Science (AP19676308) and Nazarbayev University, Astana, Kazakhstan (20122022FD4112).

## Ethics Statement

The authors have nothing to report.

## Consent

The authors have nothing to report.

## Conflicts of Interest

The authors declare no conflicts of interest.

## Supporting Information

The Supporting Information file contains additional computational analyses of ligand–protein interactions for Ag and HMB complexes with *Bacillus subtilis* target proteins (PDB IDs: 4WO7 and 2VAM), including RDG isosurface maps (Figure S1), IGM isosurface maps (Figure S2), and QTAIM topological parameters for key interactions (Table S1).

## Supporting information


**Supporting Information** Additional supporting information can be found online in the Supporting Information section.

## Data Availability

The datasets generated during and/or analyzed during the current study are available from the corresponding author upon reasonable request.
