# Supplementary material for: Ag‐Decorated Hydrogen Molybdenum Bronze Nanotubes as Dual‐Action Agents Against Bacillus subtilis: Experimental and Theoretical Insights Into Membrane Damage and Protein Interference
Source: Bioinorg Chem Appl. 2026 Feb 24;2026:9270509. doi: 10.1155/bca/9270509 (PMC12930300; doi:10.1155/bca/9270509)
Supplement: Supplementary file 1 — Supporting Information Additional supporting information can be found online in the Supporting Information section. [file BCA-2026-9270509-s001.docx]

Ag-Decorated Hydrogen Molybdenum Bronze Nanotubes as Dual-Action Agents Against *Bacillus subtilis*: Experimental and Theoretical Insights into Membrane Damage and Protein Interference

Shabnam Yavari^a,b,c^, Neda Eghtesadi^d^, Kayode Olaifa^d,e^, Darya Shafiee^f^, Amir H. Montazer^g^, Reza Rasuli^c*^, Ebrahim Nemati-Kande ^h,i^, Forough Pakzadi ^j^, Sorour Faramarzi ^h^, Mehdi Shafiee^a,b∗^

*^a^Department of Electrical and Computer Engineering, School of Engineering and Digital Sciences, Nazarbayev University, Astana, 010000, Kazakhstan*

*^b^Energetic Cosmos Laboratory, Nazarbayev University, Astana, 010000, Kazakhstan*

*^c^Department of Physics, Faculty of Science, University of Zanjan, Zanjan, Iran*

*^d^Biofilm Laboratory, Department of Chemical and Materials Engineering, School of Engineering and Digital Sciences, Nazarbayev University, Astana, 010000, Kazakhstan*

*^e^Department of Biology, Nazarbayev Intellectual School of Biology and Chemistry, Aktau, 130000, Kazakhstan.*

*^f^Department of Biomedical Sciences, School of Medicine, Nazarbayev University,*

*Astana, 010000, Kazakhstan*

*^g^Department of Medical Equipment Technology Engineering, Al-Hadba University, Mosul, Iraq.*

*^h^Department of Physical Chemistry, Faculty of Chemistry, Urmia University, Urmia 5756151818, Iran*

*^i^Research Center for Computational and Theoretical Molecular Engineering (RC2TME), Khazar University, Baku, 1009, Azerbaijan*

*^j^Department of Biology, Faculty of Science, Urmia University, Urmia 5756151818, Iran*

^∗^Corresponding authors

*Email addresses*: [r_rasuli@znu.ac.ir](mailto:r_rasuli@znu.ac.ir) (Reza Rasuli), [mehdi.shafiee@nu.edu.kz](mailto:mehdi.shafiee@nu.edu.kz)  (Mehdi Shafiee)


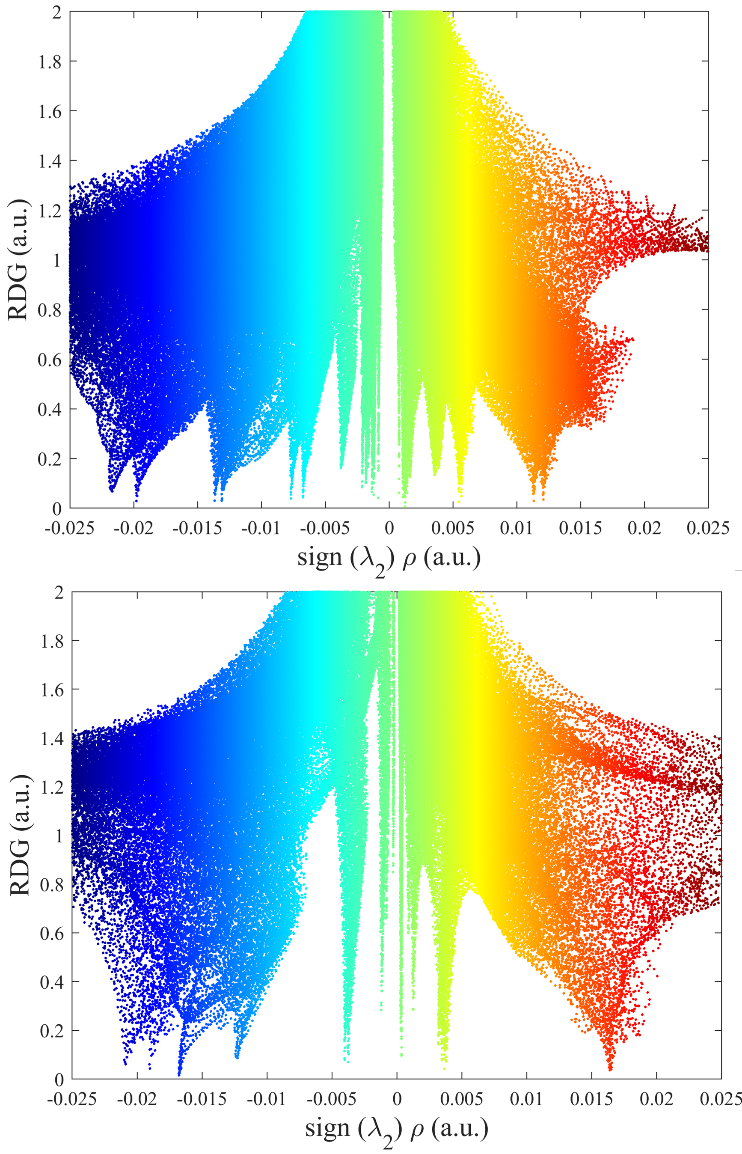

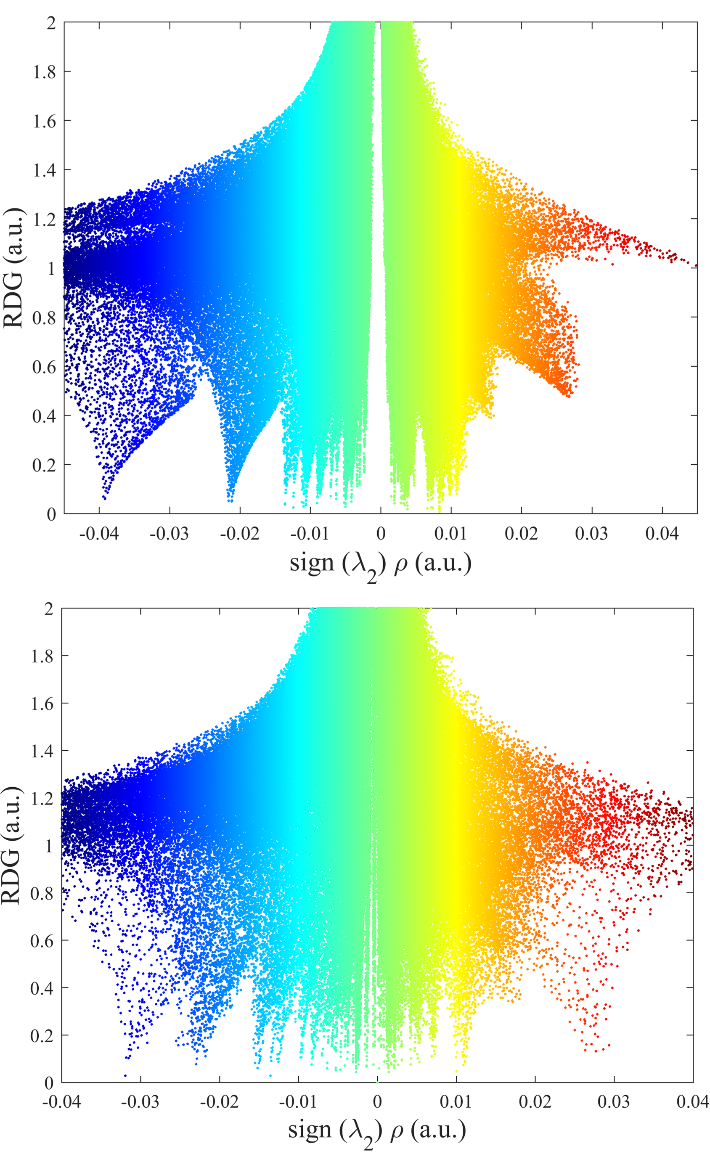


**(a)**

**(b)**

**(c)**

**(d)**

**Fig. S1:** Non-covalent interaction analysis through reduced density gradient (RDG) isosurface plots for (a,c) Ag and (b,d) HMB complexes with *Bacillus subtilis* proteins (a,b: 4WO7; c,d: 2VAM). The color scheme follows conventional RDG representation: blue regions indicate strong attractive interactions (hydrogen bonds), green areas represent van der Waals forces, and red surfaces denote steric repulsion.


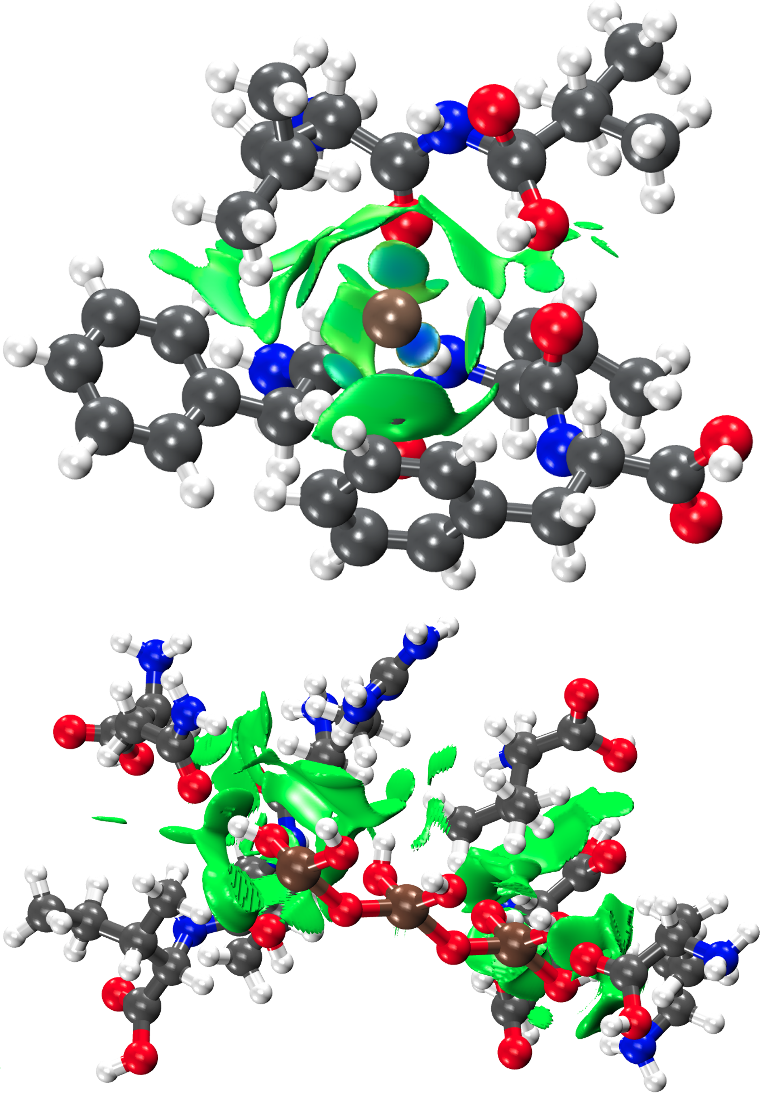

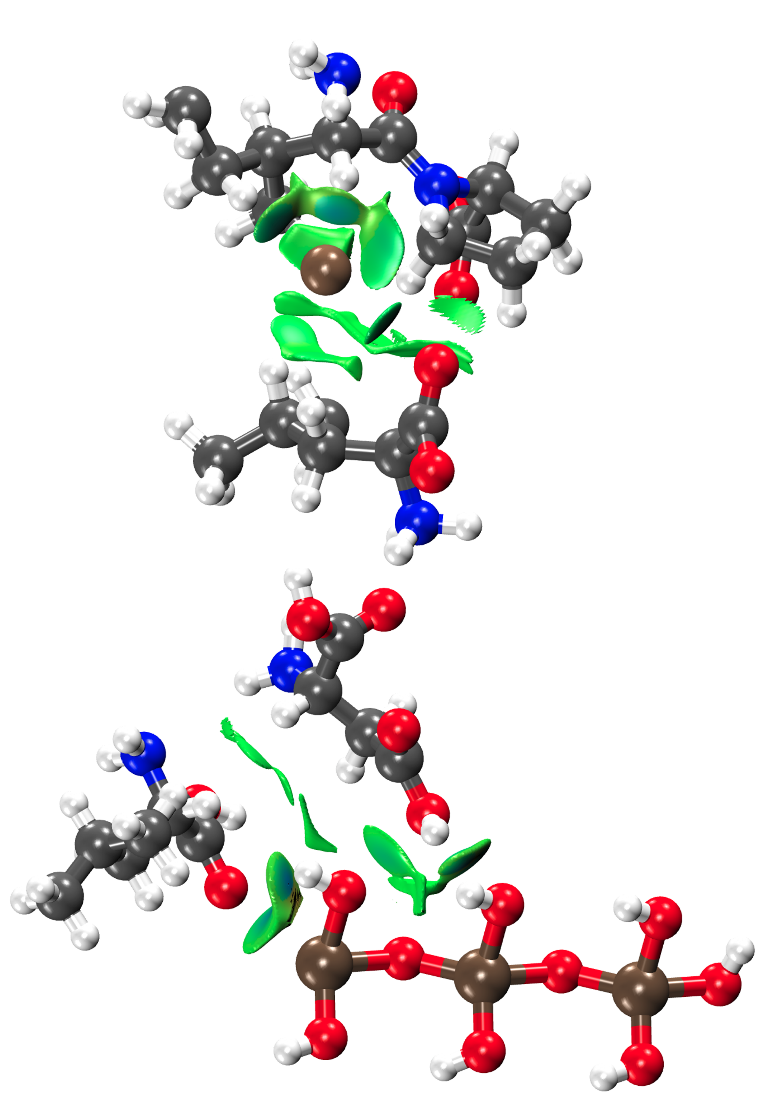


**(a)**

**(b)**

**(c)**

**(d)**

**Fig. S2:** Independent Gradient Model (IGM) isosurface analysis of non-covalent interactions between (a,c) Ag and (b,d) HMB ligands with *Bacillus subtilis* cell-wall proteins (a,b: 4WO7; c,d: 2VAM). Green isosurfaces indicate weak attractive van der Waals (vdW) interactions, where surface extent correlates with interaction strength. Isosurfaces were generated at δg = 0.03 a.u. with a promolecular density approximation, using a ±0.05 a.u. density cutoff for visualization. The isosurface value was set to 0.5 a.u. for optimal visualization of intermolecular interactions.

| ***Table S1.*** *QTAIM analysis of significant interactions between (a) Ag atoms and (b) HMB nanostructures with target proteins from Bacillus subtilis (PDB IDs: 4WO7 [cell wall protein] and 2VAM [cell cycle protein]). Key topological parameters include: R (interatomic distance, Å), ρ(r) (electron density at bond critical point, a.u.), ∇^2^ρ(r) (Laplacian of electron density, a.u.), and ε (ellipticity index, dimensionless).* | | | | | |  |
| --- | --- | --- | --- | --- | --- | --- |
| **Agent** | **Interaction** | ***R*** | ***ρ(r)*** | ∇^2^ρ(r) | **ε** |  |
| ***PDB ID: 4WO7*** | | | | | |  |
| **Ag** | **Ag ... O _(Leu)_** | *2.74* | *0.021* | 0.068 | 0.033 |  |
|  | **Ag ... H _(ILe)_** | *2.73* | *0.011* | 0.031 | 0.156 |  |
|  | **Ag ... H _(Tyr)_** | *2.36* | *0.022* | 0.055 | 0.025 |  |
|  | **Ag... HN _(Val)_** | *2.02* | *0.039* | 0.090 | 0.016 |  |
|  | **Ag … O _(Val)_** | *3.15* | *0.011* | 0.0289 | 0.377 |  |
| **HMB** | **O_(HMB)_ ... O_(Lys)_** | *2.74* | *0.013* | 0.053 | 0.879 |  |
|  | **O_(HMB)_ ... H_(Lys)_** | *2.24* | *0.015* | 0.051 | 0.058 |  |
|  | **OH_(HMB)_ ... H_(Lys)_** | *1.66* | *0.022* | 0.067 | 0.175 |  |
|  | **O_(HMB)_ ... H _(Lys)_** | *2.04* | *0.023* | 0.089 | 0.164 |  |
|  | **OH_(HMB)_ ... O_(Glu)_** | *1.84* | *0.032* | 0.143 | 0.447 |  |
|  | **OH_(HMB)_ ... O_(Val)_** | *2.06* | *0.023* | 0.071 | 0.051 |  |
|  | **OH_(HMB)_ ... H_(Arg)_** | *1.77* | *0.015* | 0.049 | 0.087 |  |
|  | **OH_(HMB)_ ...O_(Asn)_** | *2.24* | *0.016* | 0.051 | 0.017 |  |
| \| ***Ag*** \| ***Ag ... O_(Leu)_*** \| *3.00* \| *0.014* \| *0.035* \| *0.022* \| \| --- \| --- \| --- \| --- \| --- \| --- \| \| ***Ag ....H_(Pro)_*** \| *2.69* \| *0.013* \| *0.040* \| *0.291* \| \| ***Ag ...H_(ILe)_*** \| *2.397* \| *0.021* \| *0.044* \| *0.029* \| \| ***Ag ...H_(ILe)_*** \| *2.46* \| *0.019* \| *0.041* \| *0.031* \| \| ***HMB*** \| ***OH _(HMB)_ ... H_(Asp)_*** \| *1.66* \| *0.019* \| *0.074* \| *0.454* \| \| ***O_(HMB)_ ...O_(Asp)_*** \| *2.81* \| *0.012* \| *0.047* \| *0.925* \| \| ***OH_(HMB)_ ...O_(ILe)_*** \| *2.08* \| *0.021* \| *0.079* \| *0.351* \| \| ***Mo _(HMB)_ ...O_(ILe)_*** \| *2.83* \| *0.016* \| *0.055* \| *1.396* \|   ***Pdb ID: 2VAM*** | | | | | | |
